# Differential expression of paralog RNA binding proteins establishes a dynamic splicing program required for normal cerebral cortex development

**DOI:** 10.1093/nar/gkae071

**Published:** 2024-02-07

**Authors:** Eleonora Cesari, Donatella Farini, Vanessa Medici, Ingrid Ehrmann, Marika Guerra, Erika Testa, Chiara Naro, Maria Concetta Geloso, Vittoria Pagliarini, Livia La Barbera, Marcello D’Amelio, Tiziana Orsini, Stefano Farioli Vecchioli, Luca Tamagnone, Philippe Fort, Maria Teresa Viscomi, David J Elliott, Claudio Sette

**Affiliations:** Department of Neuroscience, Section of Human Anatomy, Catholic University of the Sacred Heart, Largo Francesco Vito 1, 00168 Rome, Italy; Fondazione Policlinico Agostino Gemelli IRCCS, Largo Agostino Gemelli, 00168 Rome, Italy; Department of Biomedicine and Prevention, University of Rome Tor Vergata, Via Montpellier 1, 00133 Rome, Italy; Fondazione Santa Lucia IRCCS, Via del Fosso di Fiorano, 64, 00143 Rome, Italy; Department of Neuroscience, Section of Human Anatomy, Catholic University of the Sacred Heart, Largo Francesco Vito 1, 00168 Rome, Italy; Newcastle University Centre for Cancer, Newcastle University Institute of Biosciences, Newcastle NE1 3BZ, UK; Department of Neuroscience, Section of Human Anatomy, Catholic University of the Sacred Heart, Largo Francesco Vito 1, 00168 Rome, Italy; Department of Life Science and Public Health, Section of Histology and Embryology, Catholic University of the Sacred Heart, Rome; Department of Neuroscience, Section of Human Anatomy, Catholic University of the Sacred Heart, Largo Francesco Vito 1, 00168 Rome, Italy; Fondazione Policlinico Agostino Gemelli IRCCS, Largo Agostino Gemelli, 00168 Rome, Italy; Department of Neuroscience, Section of Human Anatomy, Catholic University of the Sacred Heart, Largo Francesco Vito 1, 00168 Rome, Italy; Department of Neuroscience, Section of Human Anatomy, Catholic University of the Sacred Heart, Largo Francesco Vito 1, 00168 Rome, Italy; Fondazione Policlinico Agostino Gemelli IRCCS, Largo Agostino Gemelli, 00168 Rome, Italy; Fondazione Santa Lucia IRCCS, Via del Fosso di Fiorano, 64, 00143 Rome, Italy; Fondazione Santa Lucia IRCCS, Via del Fosso di Fiorano, 64, 00143 Rome, Italy; Università Campus Bio-Medico di Roma, Via Álvaro del Portillo, 21, 00128 Rome, Italy; Institute of Biochemistry and Cell Biology, National Research Council (IBBC/CNR), Monterotondo, 00015 Rome, Italy; Institute of Biochemistry and Cell Biology, National Research Council (IBBC/CNR), Monterotondo, 00015 Rome, Italy; Fondazione Policlinico Agostino Gemelli IRCCS, Largo Agostino Gemelli, 00168 Rome, Italy; Department of Life Science and Public Health, Section of Histology and Embryology, Catholic University of the Sacred Heart, Rome; Centre de Recherche en Biologie Cellulaire de Montpellier, University of Montpellier, CNRS, 1919 Route de Mende, 34293 Montpellier Cedex 05, France; Fondazione Policlinico Agostino Gemelli IRCCS, Largo Agostino Gemelli, 00168 Rome, Italy; Department of Life Science and Public Health, Section of Histology and Embryology, Catholic University of the Sacred Heart, Rome; Newcastle University Centre for Cancer, Newcastle University Institute of Biosciences, Newcastle NE1 3BZ, UK; Department of Neuroscience, Section of Human Anatomy, Catholic University of the Sacred Heart, Largo Francesco Vito 1, 00168 Rome, Italy; Fondazione Policlinico Agostino Gemelli IRCCS, Largo Agostino Gemelli, 00168 Rome, Italy

## Abstract

Sam68 and SLM2 are paralog RNA binding proteins (RBPs) expressed in the cerebral cortex and display similar splicing activities. However, their relative functions during cortical development are unknown. We found that these RBPs exhibit an opposite expression pattern during development. Sam68 expression declines postnatally while SLM2 increases after birth, and this developmental pattern is reinforced by hierarchical control of Sam68 expression by SLM2. Analysis of *Sam68:Slm2* double knockout (*Sam68*:*Slm2^dko^*) mice revealed hundreds of exons that respond to joint depletion of these proteins. Moreover, parallel analysis of single and double knockout cortices indicated that exons regulated mainly by SLM2 are characterized by a dynamic splicing pattern during development, whereas Sam68-dependent exons are spliced at relatively constant rates. Dynamic splicing of SLM2-sensitive exons is completely suppressed in the *Sam68*:*Slm2^dko^* developing cortex. *Sam68*:*Slm2^dko^* mice die perinatally with defects in neurogenesis and in neuronal differentiation, and develop a hydrocephalus, consistent with splicing alterations in genes related to these biological processes. Thus, our study reveals that developmental control of separate *Sam68* and *Slm2* paralog genes encoding homologous RBPs enables the orchestration of a dynamic splicing program needed for brain development and viability, while ensuring a robust redundant mechanism that supports proper cortical development.

## Graphical Abstract

**Graphical Abstract:**
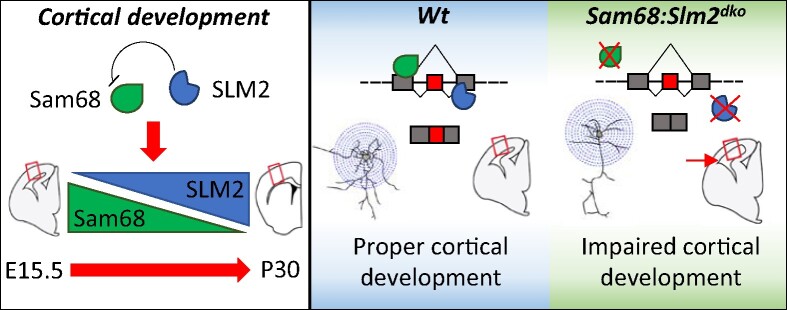

## Introduction

The mammalian brain comprises hundreds of different neurons that establish intricate neuronal networks during development. Proper achievement of this complex cellular architecture requires a tight control of the brain transcriptome, which orchestrates the timely expression of each gene in specific cell types (1–3). Furthermore, alternative splicing (AS) of neuronal genes represents an additional layer of gene expression regulation. AS is extensively modulated during brain development and several splice variants were shown to play crucial roles in the establishment of neuronal circuits or in the function of mature neurons (4,5). Splicing is the process that excises the introns from precursor messenger RNAs (pre-mRNAs) and ligates the adjacent exons to yield mature mRNAs. Several exons can be alternatively spliced to yield transcript variants that encode proteins with different structural and functional properties, thus expanding the protein repertoire of the genome (6,7). While AS occurs in almost every mammalian cell and tissue, brain was shown to express the highest number of splice variants, indicating the crucial role played by AS in this organ (4,5,8). Accordingly, several RNA binding proteins (RBPs) that regulate splicing are highly or exclusively expressed in brain (8,9), while their defective function can promote neurodegenerative and neurological diseases (5,8–10).

Developmental control of AS ensures the fine-tuned modulation of protein isoforms that mediate synaptic contacts and, consequently, synaptic function and plasticity (11–13). In this regard, the *Neurexin* gene family (*Nrxn1-3*) is a prototypical example (14). NRXN1-3 are presynaptic adhesion proteins that regulate synapse specification and functions by interacting with postsynaptic receptors (14,15). NRXN splice variants induce clustering of different proteins in the postsynaptic compartment, thus adapting the functional properties of the synapse to cues (15). The distribution of NRXNs in excitatory or inhibitory synapses, as well as their interaction with specific ligands, are tightly regulated by AS (14,15). Indeed, regulation of the alternatively spliced segment 4 (AS4) is modulated by neuronal activity (16–18) and inclusion/skipping of this exon determines the choice between postsynaptic N-methyl-D-aspartate (NMDA) or α-amino-3-hydroxy-5-methyl-4-isoxazolepropionic acid (AMPA) receptors (19). This splicing switch directly affects synaptic responses (19) and its dysregulation *in vivo* impairs cognitive functions (17,20,21), further highlighting the key role played by AS in the control of neuronal circuitries.

The interplay between paralog RBPs can contribute to AS regulation during brain development. For instance, the homologous polypyrimidine tract binding proteins 1 (PTBP1) and 2 (PTBP2) are expressed, respectively, in neural progenitor cells (NPCs) and in committed neurons at the onset of differentiation (22). In NPCs, PTBP1 represses the expression of PTBP2 to maintain a stem-like phenotype. PTBP1 downregulation at the onset of neurogenesis unleashes PTBP2 expression, setting in motion a neuron-specific splicing program (5). Thus, although these paralog RBPs share many structural and functional features (5), with high redundancy in splicing control, their differential expression allows fine-tuned control of an important splicing program during neurogenesis. Another family of RBPs with important roles in brain is the Signal Transduction and Activation of RNA (STAR) family (23). In particular, the Sam68 subfamily of STAR proteins, comprising Sam68 (Src-associated protein in mitosis of 68 kDa, KHDRBS1), SLM1 (Sam68-like mammalian protein 1, KHDRBS2) and SLM2 (Sam68-like mammalian protein 2, KHDRBS3), were shown to govern splicing of *Nrxn1-3* and other synaptic genes (16,18,24). While Sam68 is expressed in most cells of brain and other organs (25), SLM1 and SLM2 display a cell- and tissue-specific pattern and their expression is particularly enriched in neurons (23,24,26,27). Accordingly, knockout of Sam68 affects multiple organs and processes, including bone and fat homeostasis (25,28), male and female fertility (29,30) and liver gluconeogenesis (31). Sam68 is also highly expressed in brain, particularly in the olfactory bulb and cerebellum (18,26,32), and its ablation in mice impairs fetal neurogenesis (32), motor coordination (16,18,33), glutamatergic synapses (18,34) and social behaviour (18). By contrast, ablation of *Slm1* expression did not cause noticeable defects (26), while *Slm2* knockout specifically impaired hippocampal circuits involved in spatial memory (20,21). Distinct *Sam68* and *Slm2* genes have been maintained for > 500 million years, despite these proteins having similar target RNA sequences and jointly regulating many target genes. However why maintenance of these two genes has been evolutionary important is unknown, nor is known how Sam68 and SLM2 interact with each other during embryonic and post-natal brain development to ensure proper temporal splicing patterns.

In line with its pleiotropic functions in the brain, Sam68 regulates AS of multiple genes encoding for proteins that play a key role in the brain (18,32,35). On the other hand, very few target genes were identified for both SLM1 and SLM2 (20,21,35) and most of them are also targets of Sam68. The only clear exception is the AS4 exon of *Nrxn2*, which is exclusively regulated by SLM2 (24). This specificity was related to the low density of binding sites for these RBPs flanking the *Nrxn2* AS4 exon, which reduced the affinity for Sam68 but not for SLM2 (36). Nevertheless, it is unlikely that the *Slm2* paralog gene has evolved to control a single exon in the genome. To investigate why it is necessary to have separate Sam68 and SLM2 proteins, we have generated mice that are knocked out individually and jointly for the *Sam68* and *Slm2* genes. Our study indicates that SLM2 expression progressively increases during cortical development, where its expression pattern controls the dynamic regulation of a select group of exons during development. Moreover, by comparing single and double knockout mice, we find that concomitant ablation of these paralog RBPs exacerbates previously undetected defects of the single knockout mice in neurogenesis, synaptic organization, neuron morphology and viability, showing that this dynamically regulated joint function of Sam68 and SLM2 plays an important role in the developing cortex.

## Materials and methods

### Mouse husbandry

C57BL/6 mice wild type, *Sam68^ko^*, *Slm2^ko^* and *Sam68:Slm2^dko^* mice were maintained on a normal 12 hr light/dark cycle in the animal facility of the Fondazione Santa Lucia IRCCS and genotyped by the Biotool Mouse Direct PCR Kit. Mice breeding and housing were conducted according to the Guideline of the Italian Institute of Health (protocol no. 157/2019-PR).

### Primary cortical cell culture

Cortical neurons cultures were prepared as previously reported (37) from both male and female C57BL/6 mouse pups on embryonal day (E) 14.5. Tissue was dissociated with 0.025% trypsin (Sigma-Aldrich) and neurons were plated into poly-D-lysine-coated plates and maintained for 5 (DIV5) or 14 days (DIV 14) in Neurobasal Medium (Invitrogen) containing 2% B27 supplement, 2 mM glutamine, gentamycin (100 mg/ml) and 50 U penicillin/streptomycin (GIBCO). Neurons were fed by exchanging half of the media every 3–4 days.

### RNA isolation and RT-PCR

RNA was extracted from cortex using Trizol reagent (Invitrogen) and retro-transcribed (1 μg) with oligo-dT oligonucleotides and M-MLV reverse transcriptase (Promega). 15 ng of cDNA was used as template for PCR (GoTaq, Promega) and reactions were analyzed on agarose gels. Quantitative real-time PCRs (qPCR) were performed using LightCycler 480 SYBR Green I Master and the LightCycler 480 System (Roche). Control reactions omitting M-MLV reverse transcriptase were also carried out. Sam68/SLM2 splice variants were detected by RT-PCR using previously described specific primers and capillary gel electrophoresis (21). Reactions were quantitated by calculating the percentage splicing inclusion (PSI) as described (21). All PCR primers are listed in the Supplementary Table S1.

### RNA-Seq and bioinformatics analyses

Cortices from wild-type and *Sam68:Slm2^dko^* E18.5 embryos were isolated and maintained in RNA later stabilization reagent (QIAGEN). Total RNA was extracted and DNase treated using the RNAeasy Mini Kit (QIAGEN) according to manufacturer's instruction. PolyA plus RNA-seq libraries were constructed and sequenced using a 150 bp paired-end format on an Illumina NovaSeq6000. RNA-seq data analysis was performed by GenoSplice technology as previously described (38,39). Gene Ontology (GO) Enrichment analyses for Biological Process and Cellular Component were performed using Cluster Profiler package in R Studio Software. All analyses were performed using a background consisting of 12 322 expressed genes using the following parameters: 0.05 p-value, 0.2 q-value and Benjamini Hochberg False Discovery Rate (FDR) as p-Adjusted Method.

### Protein extracts and western blot analysis

Cortices from wild-type and knockout mice were homogenized in RIPA buffer [50 mM Tris pH 7.4; 1% NP-40; 0.5% Na deoxycholate; 0.1% SDS; 150 mM NaCl; 1 mM EDTA; 1 mM DTT; 0,5 mM NaVO3; protease inhibitor cocktail (Sigma Aldrich)]. Lysates were incubated on ice for 30 min, briefly sonicated, and centrifuged (10 min at 13 000 rpm, 4°C). Protein extracts were quantified by Bradford assays and analyzed by Western Blot using the following primary antibodies: rabbit anti-Sam68 (dilution 1:2000, Bethyl Laboratories), rabbit anti-SLM2 (dilution 1:1000, Sigma-Aldrich), mouse anti-ACTIN (dilution 1:1000, Santa Cruz Biotechnology). Coomassie staining was used as loading control. Anti-rabbit, anti-mouse HRP-linked secondary antibodies (GE Healthcare) were used at 1:5000 dilution and ECL signal developed using Clarity Western ECL Blotting Substrate (Biorad).

### Immunofluorescence analysis

Starting from P10, mice were perfused transcardially with 50 ml of saline, followed by 50 ml of 4% paraformaldehyde (PFA) under anesthesia, which was induced by intraperitoneal injections of Rompun (xylazine, 20 mg/ml, 0.5 ml/kg body weight) and Zoletil (tiletamine and zolazepam, 100 and 0.5 ml/kg body weight). All wild-type male brains were dissected, fixed in 4% paraformaldehyde for 1 day and, after 3 washes in PBS, transferred to a 30% sucrose solution at 4°C until they sank. Brains were cut into 6 series of 40 μm-thick transverse sections using a cryostat, and the slices were collected in PBS. Brain sections were incubated overnight at 4°C in PBS containing 0.3% Triton X-100, the following primary antibodies: rabbit anti-Sam68 (1:400), rabbit anti-SLM2 (1:200); mouse anti-CTIP2 (Abcam; 1:200); goat anti-Iba-1 (Novus Biologicals; 1:600); mouse anti-NeuN (Sigma-Aldrich; 1:200); mouse anti-GFAP (Santa Cruz Biotechnology; 1:200); mouse anti CNPase (Abcam; 1:400). After 3 washes in PBS, the sections were incubated for 2 h at room temperature with a cocktail of secondary antibodies, including Alexa Fluor 488 donkey anti-mouse IgG or Alexa Fluor 647 donkey anti-mouse IgG, Alexa Fluor 555 donkey anti-rabbit IgG (1:200; Invitrogen). The sections were counterstained with Neuro-Trace® 640/660 deep-red Fluorescent Nissl Stain (1:200; Invitrogen). After three washes in PBS, sections were mounted using an anti-fade medium (Fluoromount; Sigma-Aldrich).

For VGluT1 staining, cortical neurons were fixed in 4% PFA for 15 min, permeabilized in 0.1% Triton for 10 min and incubated 1 h in blocking buffer solution containing 0.5% BSA and 5% goat serum diluted in PBS. Cells were then incubated with guinea pig anti-VGluT1 (1:800; Synaptic System) and Alexa Fluor 488 phalloidin 488 (1:600; Cell Signaling) in blocking solution at 4°C overnight. Following rinsing, samples were incubated with donkey anti-guinea pig Alexa Fluor 555 secondary antibody (1:200) for 2 h. All Image acquisitions were performed by using a confocal laser-scanning microscope (CLSM Leica SP5). All samples were captured using consistent settings for laser power and detector gain. Images were generated by adjusting only the brightness and the contrast and composed using Adobe Illustrator CS5. Quantification of VGluT1 puncta density was performed by using Fiji software (version 2; National Institute of Health, USA). Photomicrographs were converted to 8-bit images and the automated thresholding algorithm Intermodes was applied. Subsequently, the ‘‘Watershed’ option was applied to separate puncta that appeared fused. VGluT1 puncta density counts were determined using the ‘‘Analyze particles’ option in two squared frames (400 μm^2^) pseudo randomly distributed on cell body and neuronal processes of each phalloidin-labelled neuron considered (*n* = 50 wt; *n* = 53 *Sam68^ko^*; *n* = 40 *Slm2^ko^*; *n* = 40 *Sam68:Slm2^dko^*; *n* = 5 mice for experimental group). Data were collected by two independent blind researchers.

### Sholl's analysis

Morphological analysis of cortical neurons was performed on 5 days *in vitro* (DIV 5) cultures. Neurons were fixed in 4% PFA and immunostained with mouse anti-β3 tubulin (Millipore AB9354) and counterstained with DAPI and images were acquired with an optical microscope (DMLB; Leica) equipped with a motorized stage and a camera connected to Neurolucida 7.5 software (MicroBright-Field) for quantitative 3D analysis of the entire cell compartment. Only neurons showing intact processes were included in cell reconstructions. To account for changes in cellular complexity in relation to distance from the soma, concentric circles (radii) were spaced 10 μm, starting from the soma. The number of branch points and endings, processes that intersected the radii and process length were measured as a function of the distance from the cell soma for each radius. For morphological analyses 7–10 neurons per culture were randomly selected (*n* = 4 mice for each experimental group). Data were collected by two independent blind researchers and averaged for each culture.

### RNAscope

Mice were transcardially perfused as above and 20 μm coronal sections were mounted onto Superfrost Plus slides (Thermo Fisher Scientific, Pittsburgh, PA). RNAscope in combination with immunofluorescence was performed by using RNAscope Multiplex Fluorescent Detection Reagents v2 (cat#323110; Advance Cell Diagnostics, Hayward, CA) and the mouse SLM2 probe (Mm-Khdrbs3 cat# 496551; Advance Cell Diagnostics, Hayward, CA). Sections were first incubated with primary antibodies (anti-Sam68, anti-SLM2) and then processed for RNAscope. At the end of the procedure, sections were incubated for 1 h at room temperature with Alexa Fluor 488 donkey anti-rabbit (1:200; Invitrogen) and Dapi-counterstained. Images were taken by using Nikon Eclipse Ti1 confocal microscope. Quantitative analysis of Sam68/SLM2 positive cells or SLM2 antibody/SLM2-RNAscope positive cells was performed on digital images. All double and single labelled cells in two digital squared frames (400 μm per side) in five brain sections, regularly spaced throughout the caudo-rostral extent of the cerebral cortex, were counted (5 sections/mouse; *n* = 3 mice). Double and single immunolabeled cells were then digitally marked, counted and analyzed.

### String analysis

Differentially regulated genes for AS in DKO belonging to cell cycle and synapse GO categories, were used as input for String analysis (40). The minimum required interaction score was based on medium confidence (0.400).

### Micro computed tomography analysis

E18.5 embryos were fixed in 4% PFA and then immersed in a potassium iodine contrast agent, 0.1N (v/v) Lugol solution (Sigma-Aldrich), at room temperature for 2 weeks, replacing it several times during the treatment. Tomographic datasets were generated by a high-resolution 3D micro-CT imaging system (Skyscan 1172G Bruker, Kontich – Belgium), using a L7901-20 Microfocus X-ray Source (Hamamatsu). Acquisitions were performed in 5 ml plastic tubes, setting a camera pixel/size of 10.9 μm, binning 2 × 2, tube voltage peak of 39 kV, tube current of 240 μA, exposure time of 370 ms. Reconstructions of virtual samples were performed using built-in NRecon Skyscan Software (Version: 1.6.6.0; Bruker), while 3D images were generated using 3D Visualization Software CTvox v. 2.5 (Bruker). Manual image-by-image segmentation aimed at volumes calculation was applied by Bruker micro-CT Analyser Version 1.13 software, using the histological atlas of mouse development as guidance to accurately identify, demarcate and segment each sample (*n* = 6) in a specific Volume of Interest (VOI) for automated volume measurements. The ventricle volume of each region was estimated using the following formula: *V* = *Ta*Σ*Pi*, where *T* is the mean slice thickness, *a* is the area per point and Σ*Pi* is the sum of points hitting the marked region. Coefficients of error were calculated, and only values <0.1 were accepted. For the estimation of the lateral ventricle volume in adult brain, the Cavalieri Estimator Probe method was used (41). Stereological analysis was conducted in 6 serial 30 μm thick coronal sections per mouse (wild-type *n* = 4, dKO *n* = 4;) using the Stereo-Investigator software package (Stereo Investigator software, Version 9, MicroBrightField Europe, Magdeburg, Germany) and a Nikon Eclipse 80i digital photomicroscope.

### Quantification and statistical analysis

Data are presented as mean ± standard error (SE), *n* ≥ 3, unless otherwise indicated. All tests were performed using GraphPad Prism6. The statistical tests used are specified in each Figure legend. Student's t-test was applied for comparisons between two groups; one-way and two-way ANOVA tests were applied for comparisons between multiple samples. *P*-value <0.05 was considered significant. The machine learning algorithm analysis self-organizing maps was undertaken using the ‘som’ R package with topol = ‘hexa’, neigh = ‘Gaussian’.

## Results

### SLM2 expression is developmentally regulated in the mouse cortex

Sam68 is almost ubiquitously expressed in the adult mouse brain (26), whereas SLM2 expression is regionalized with a rostro-caudal gradient (24), being high in the cortex and CA subfields of the hippocampus and minimal in the cerebellum (18,24). Both RBPs are expressed in the adult cortex (26). However, while cortical expression of Sam68 was shown to peak between embryonic day 13.5 (E13.5) and E15.5, and to gradually decrease after birth (32), the developmental pattern of SLM2 expression is unknown. To investigate this issue, we performed immunofluorescence and confocal analysis of the mouse cortex at four developmental stages: E15.5, when neuronal precursors proliferate and the first post-mitotic neurons stratify in the cortical layers; postnatal day 0 (P0 or birth), when post-mitotic neurons migrate towards the pial surface; P10, at the peak of the cortical lamination and active synaptogenesis; and P30, when synaptic maturation is refined and cortical development is completed (42).

**Figure 1.**
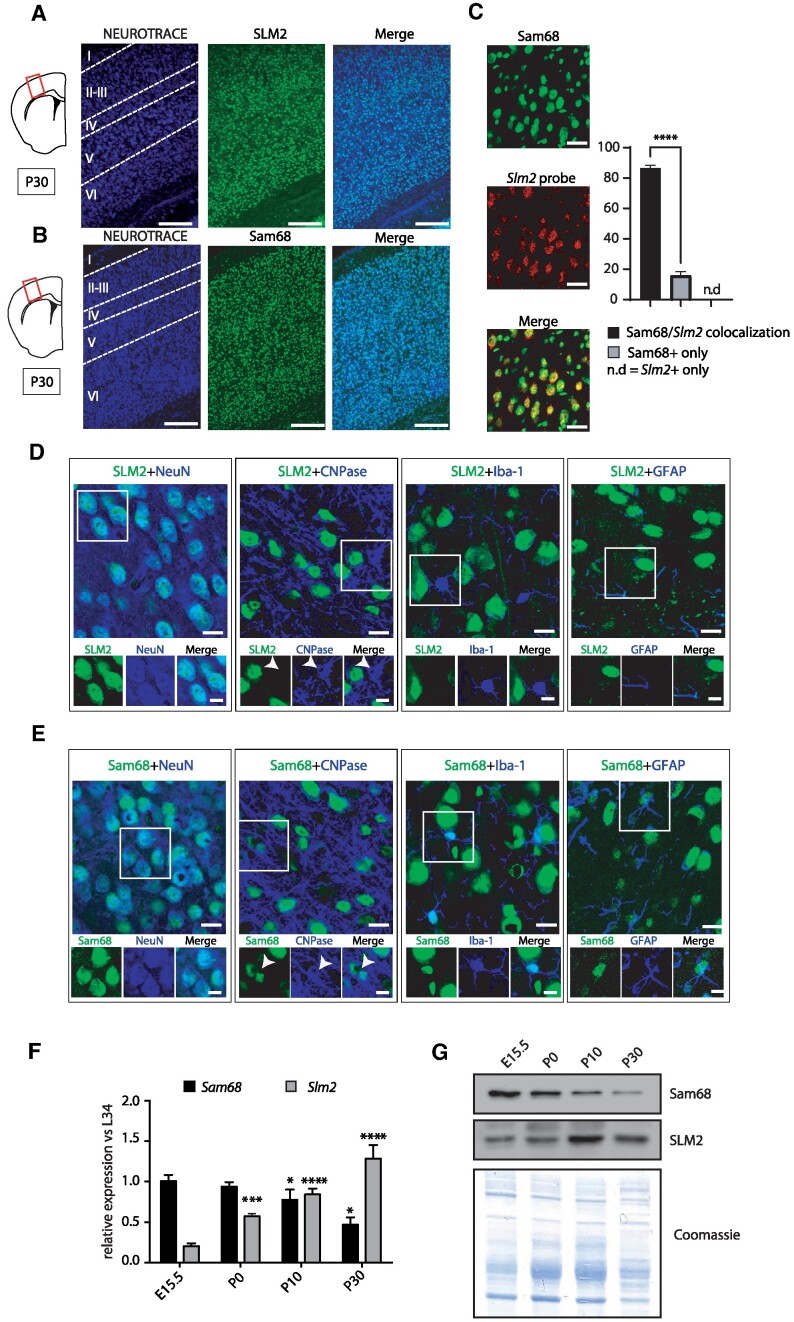
SLM2 cortical expression steadily increases during development. (A, B) Confocal images of SLM2 (**A**) or Sam68 (**B**) staining (green) counterstained with Neurotrace to show all brain cells (blue) in the mouse cortex at P30. The cortical regions corresponding to layers I-VI are marked by dotted lines in the Neurotrace image. A scheme of the cortical region examined is shown on the left of the panels. Scale bar 50μm. (**C**) Confocal images of Sam68 and *Slm2* mRNA (RNAscope probe). The bar graph represents the percentage of cells displaying Sam68 and *Slm2* colocalization or stained by only one of the two. Scale bar 20 μm. Data represent the mean + standard error (SE) of at least three independent samples. Statistical analysis was performed independently for Sam68 and Slm2 probe expression by one way ANOVA Tukey's multiple comparisons test; ****P= 0.0001. (D, E) Confocal images of SLM2 (**D**) or Sam68 (**E**) and NeuN, CNPase, Iba1 or GFAP in the layer V of the sensory motor cortex. Scale bars 20 μm. (F, G) Quantitative q-PCR (**F**) and Western blot (**G**) analyses of Sam68 and SLM2 transcript and protein expression in the developing cortex. L34 was used for normalization of the q-PCR data (F). Data represent the mean + standard error (SE) of at least three independent samples. Statistical analysis was performed independently for Sam68 and SLM2 expression by one way ANOVA Tukey's multiple comparisons test; *P= 0.05, ***P= 0.001, ****P= 0.0001. Coomassie blue staining was performed as loading control of the western blot (G).

At E15.5, when only the innermost cortical layers have formed (43), SLM2 expression was high in the cortical plate (CP), with dimmer staining being also detected in the neurogenic ventricular (VZ) and subventricular (SVZ) zones (Supplementary Figure S1A, B). At P0, when the cortical plate begins to expand and the upper layers are being formed, SLM2 was more widely distributed (Supplementary Figure S1A), with high expression in layer V that, at this developmental stage, is marked by the transcription factor CTIP2 (44). Post-natal expression (P10–P30) of SLM2 was also distributed in the whole cortex (Figure 1A; Supplementary Figure S1A), with a slight gradient from the innermost layers VI and V (higher) to the outer Layers II-III (lower) detected at P10 (Supplementary Figure S1A,C). Interestingly, an opposite gradient was observed for Sam68 at P10, with this RBP being expressed at higher levels in the outer than in the innermost cortical layers (Supplementary Figure S1C, D). Noteworthy, both RBPs were widely expressed in the adult (P30) cortex (Figure 1A, B). Co-staining with a RNA probe for SLM2, which largely recapitulated the expression of the SLM2 protein (Supplementary Figure S1E), and the anti-Sam68 antibody showed that the two RBPs were co-expressed in most cortical cells (>80%) at this stage of development, while the remaining ones only expressed Sam68 (Figure 1C). To further characterize the cell types expressing Sam68 and SLM2, P30 cortical sections were co-stained with neuronal (NeuN), oligodendrocyte (CNPase), microglia (IBA) and astrocyte (GFAP) markers. SLM2 co-localized exclusively with the neuronal marker NeuN (Figure 1D). By contrast, Sam68 is co-localized with all these markers, albeit its expression in neurons was higher than in the other cell types (Figure 1E). Moreover, real-time quantitative PCR (qPCR) and western blot analyses indicated that SLM2 transcript and protein levels increased in the developing cortex from E15.5 to P30, whereas Sam68 expression was significantly reduced after birth (Figure 1F, G). These findings show that these paralog RBPs exhibit an opposite expression pattern during cortical development.

### SLM2 controls the expression of Sam68 in the postnatal cortex

**Figure 2.**
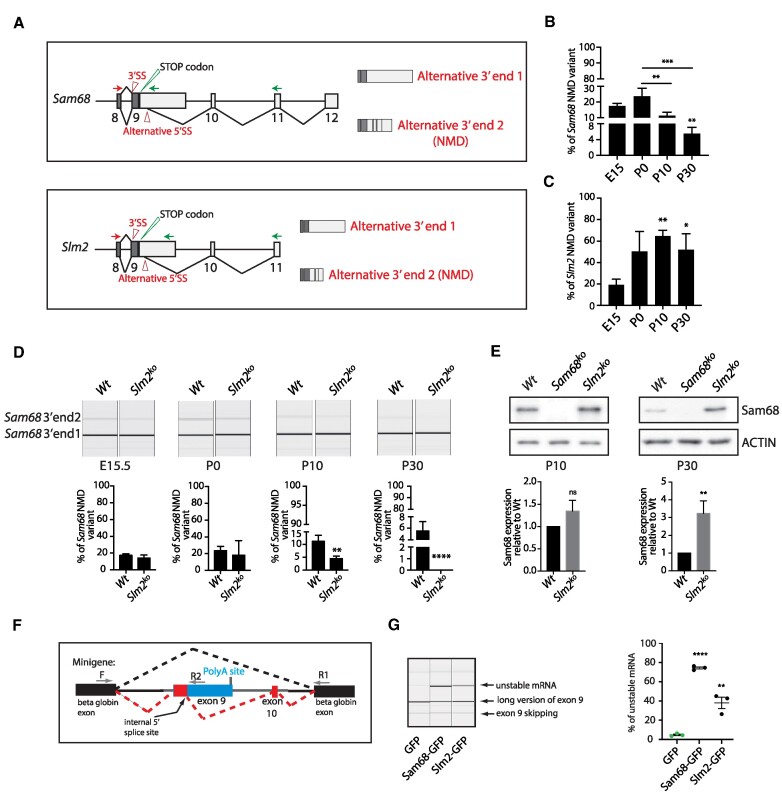
SLM2 represses the expression of Sam68 in the adult cortex. (**A**) Schematic representation of the canonical full-length and the NMD-targeted splice variants of *Sam68* (upper panel) and *Slm2* (lower panel) genes. The position of the forward (red) and reverse (green) primers used for detection of the alternative end variants 1 and 2 by capillary RT-PCR analyses are indicated by arrows. (B, C) Quantitative capillary RT-PCR analyses of the percentage of NMD-targeted alternative end 2 mRNA variant of *Sam68* (**B**) and *Slm2* (**C**) in the wild-type cortex at the indicated developmental stages. (**D**) Quantitative capillary RT-PCR analyses of the percentage of NMD-targeted alternative end 2 mRNA variant of *Sam68* in the wild-type and *Slm2^ko^* cortex at the indicated developmental stages. (B–D) Data are expressed as alternative end 2/alternative end 1 + alternative end 2 and represent the mean + SE of at least three independent samples. Statistical analysis was performed by one way ANOVA with respect to the E15.5 ratio for each gene; *P= 0.05, **P= 0.01, ***P= 0.001, ****P= 0.0001. (**E**) Representative Western blot analysis of Sam68 protein expression in the wild-type and *Slm2^ko^* cortex at P10 and P30. Bar graphs show the densitometric analysis of three independent samples. Statistical analysis was performed by Student's *t*-test; **P= 0.01, n.s.= not significant. (**F**) Schematic representation of the minigene encompassing the *Sam68* genomic region between the canonical exon 9 and the NMD-associated exon 10. The different alternative splicing options are show by dotted lines. (**G**) Quantitative capillary RT-PCR analyses of the percentage of unstable mRNA in HEK293T cells transfected with the *Sam68* minigene and Sam68-GFP or SLM2-GFP plasmid. Statistical analysis was performed by Student's *t*-test respect to control (GFP); **P= 0.01, ****P= 0.0001.

SLM2 and Sam68 self-regulate their own expression through a non-sense mediated decay (NMD) mechanism, which involves usage of a cryptic 5′ splice site in the terminal exon 9 and splicing of additional non-coding downstream exons (21). Due to the presence of a stop codon in the proximal part of exon 9, inclusion of these distal exons targets to NMD the SLM2 and Sam68 transcripts ending at the alternative end 2 (Figure 2A), thus preventing their translation into protein. This mechanism is thought to limit the over-production of these RBPs, which has been associated with neoplastic transformation (45,46). However, these studies were only performed in adult mice. To assess whether Sam68 and SLM2 expression undergo NMD-associated autoregulation during cortical development, when their relative expression varies, we analyzed the expression of their alternative transcripts at various ages. Isoform specific RT-PCR followed by capillary gel electrophoresis indicated that the NMD-associated variants of Sam68 and SLM2 exhibit opposite trends, with the Sam68 end 2 transcript declining and the SLM2 end 2 transcript increasing during cortical development (Figure 2B, C). This result indicates that NMD-directed regulation of both RBPs is maximal when their respective expression is higher in the cortex (Figure 1F,G). Next, we evaluated whether Sam68 and SLM2 regulate each other during cortical development by analyzing the expression of the NMD-associated transcripts of both genes in mice that are knocked out for either *Slm2* (24) or *Sam68* (25). The *Sam68* NMD-associated transcript was not affected by ablation of the *Slm2* gene at developmental stages (E15.5 and P0) when SLM2 is expressed at low levels. However, concomitant with the up-regulation of SLM2 protein in wild-type mice (Figure 1G), the Sam68 alternative end 2 transcript was significantly reduced in the *Slm2^−/−^* cortex at P10 and it was almost undetectable at P30 (Figure 2D). This effect on the splicing of the canonical last exon was correlated with a progressive increase in Sam68 protein levels in the *Slm2^−/−^* cortex between P10 and P30 (Figure 2E). By contrast, splicing of the *Slm2* NMD-associated variant was not altered by *Sam68* ablation (Supplementary Figure S2A). Indeed, the mild reduction of the *Slm2* alternative end 2 transcript in the P10 *Sam68^−/-^*cortex (Supplementary Figure S2A) was not accompanied by significant changes at the protein level (Supplementary Figure S2B,C).

To confirm the regulation of Sam68 splicing by SLM2, we generated a minigene encompassing its genomic region between the canonical exon 9 and the NMD-associated exon 10 (Figure 2F). Co-transfection of the Sam68 minigene with a GFP-encoding plasmid prevalently yielded the canonical splice variant with the full exon 9 included. However, when we co-transfected it with SLM2-GFP, or GFP-Sam68 as positive control (21), we observed inclusion of exon 10 indicating expression of the unstable, NMD-associated variant of Sam68 (Figure 2G). This orthogonal assay confirms that SLM2 protein can modulate the expression of Sam68 through NMD-associated splicing. Thus, increased expression SLM2 after birth contributes to limit the expression of Sam68 and to the switch in the relative expression of these paralog RBPs during cortical development.

### Splicing of joint Sam68 and SLM2 target genes is dynamically regulated during cortical development

**Figure 3.**
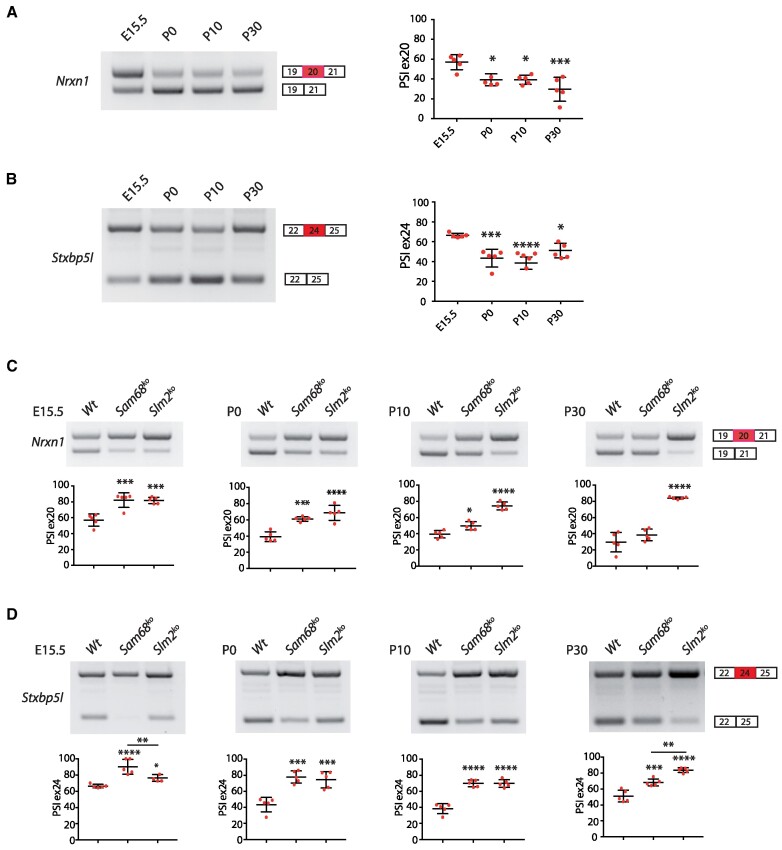
Dynamic splicing regulation of common exon targets of Sam68 and SLM2 during cortical development. (A, B) RT-PCR analyses of the *Nrxn1* exon 20 (**A**) and *Stxbp5l* exon 24 (**B**) splicing pattern in the wild-type cortex isolated at the indicated developmental ages. Graphs report the densitometric analysis of the ‘percent spliced in’ (PSI) of the alternative exon and represent the mean ± SE of five independent samples. (C, D) RT-PCR analyses of the *Nrxn1* exon 20 (**C**) and *Stxbp5l* exon 24 (**D**) splicing pattern in the wild-type, *Sam68^ko^* and *Slm2^ko^* cortices isolated at the indicated developmental ages. Graphs report the densitometric analysis of the PSI of the alternative exon and represent the mean ± SE of five independent samples. (A–D) Statistical analyses were performed with one-way ANOVA, Tukey's multiple comparisons test; *P < 0.05; **P < 0.01***P < 0.001; ****P < 0.0001.

Sam68 and SLM2 are highly homologous and display similar splicing activities *in vitro*. Nevertheless, some targets appear to be unique for either protein. For instance, the AS4 exon (exon 22) of *Nrxn2* is specifically repressed by SLM2, whereas the homologous exon in *Nrxn1* (exon 20) is controlled by both Sam68 and SLM2 (16,24). Overall, SLM2 has a selective group of splicing targets (20,21), while Sam68 controls the splicing of hundreds of exons in brain (18,32,47). Hence, we hypothesized that the switch in expression of Sam68 and SLM2 during cortical development could be associated with dynamic splicing regulation of either their common (*Nrxn1* and *Stxbp5l*) or unique (*Nrxn2* and *Sgce*) targets. Supporting this hypothesis, semi-quantitative RT-PCR (sqPCR) analyses showed a dynamic splicing regulation of commonly regulated exons. Skipping of *Nrxn1* exon 20 showed a clear switch between E15.5 and P0, that was maintained until P30 (Figure 3A). Moreover, inclusion of the *Stxbp5l* exon 24 decreased between P0 and P10 and it was partially rescued at P30 (Figure 3B). Developmental-regulated splicing was also observed for the SLM2-specific *Nrxn2* gene, with a clear switch toward skipping at birth (Supplementary Figure S3A). Conversely, splicing of the Sam68-specific *Sgce* exon 9 was relatively unchanged from E15.5 to P30 (Supplementary Figure S3B). Importantly, the dynamic splicing pattern of the *Nrxn1/2* and *Stxbp5l* genes in the developing cortex was recapitulated by differentiation of cortical neurons for increasing days *in vitro* (DIV0-DIV14; Supplementary Figure S3C–E), indicating that it was associated with maturation of neurons during development.

Since Sam68 and SLM2 display an opposite trend of expression during cortical development (Figure 1F,G), we hypothesized that these paralog RBPs might have different levels of importance for splicing control of common targets at different ages. To test this hypothesis, we analyzed the splicing patterns within the cortex of *Sam68^−/-^*and *Slm2^−/-^*mice during development. Consistent with its higher expression before birth, knockout of *Sam68* significantly impaired skipping of *Nrxn1* exon 20 at E15.5 and P0, while it exerted mild (P10) or no effects (P30) at later stages (Figure 3C). By contrast, knockout of *Slm2* impaired exon 20 skipping throughout cortical development. However, the effect increased after birth and knockout of *Slm2* expression almost completely rescued exon 20 inclusion at P30 (percent-spliced-in, PSI, >80%; Figure 3C). An inverse gradient of the impact of these STAR proteins on splicing was also observed for *Stxbp5l*. In this gene, exon 24 was almost completely under the control of Sam68 at E15.5, which continued to exert a reduced but still significant effect also at P30. By contrast, knockout of *Slm2* had a marginal effect on cortical patterns of *Stxbp5l* splicing at E15.5 but had the predominant effect at P30 (Figure 3D). Importantly, a similar time-associated dependency on Sam68 (early stage) or SLM2 (late stage) was also recapitulated in cortical neurons differentiated *in vitro* (Supplementary Figure S3F, G), even though the extent of switch was not as evident as in the P30 cortex. Together, these results are consistent with Sam68 and SLM2 having specific individual roles in splicing control of common targets during pre- and postnatal development, with Sam68 playing a more relevant role in the early stages of cortical development and SLM2 taking control after birth.

### Combined ablation of Sam68 and SLM2 expression causes perinatal lethality

**Figure 4.**
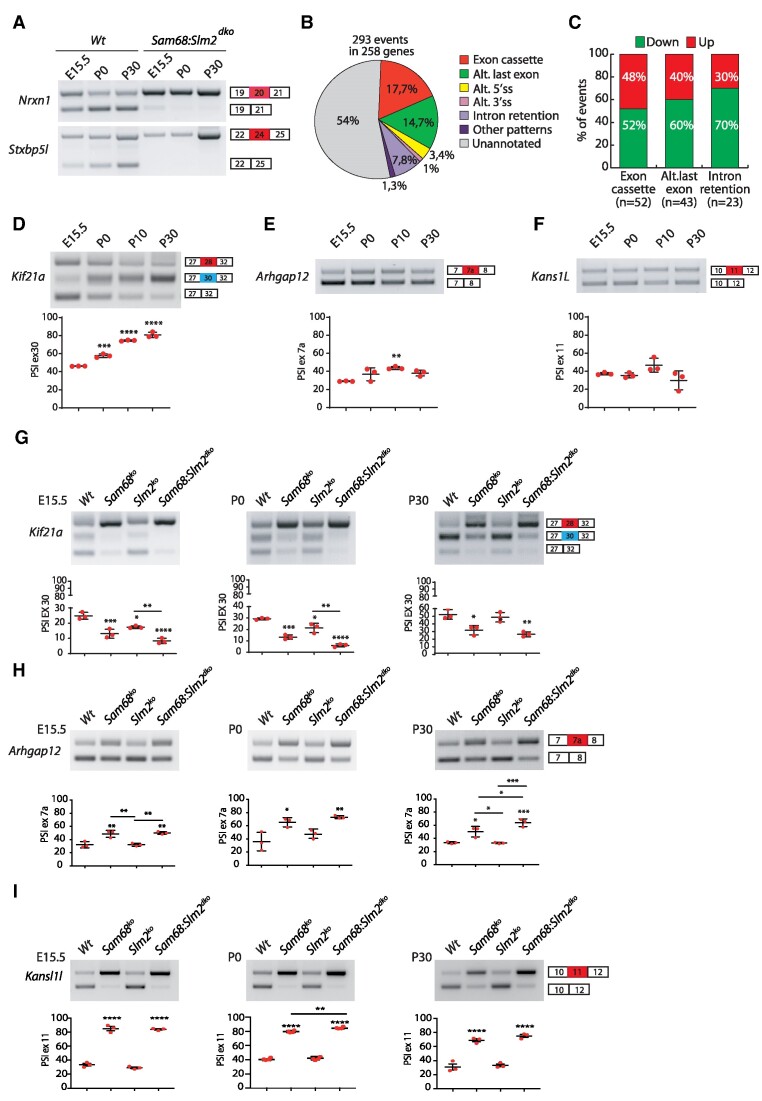
Sam68 and SLM2 regulate a widespread splicing program in the developing cortex. (**A**) Representative RT-PCR analysis of the splicing pattern of *Nrxn1* exon 20 and *Stxbp5l* exon 24 during development of the wild-type and *Sam68:Slm2^dko^* cortex. (**B**) Pie chart of the splicing events and splicing patterns that are differentially regulated in the *Sam68:Slm2^dko^* cortex. (**C**) Bar graph representation of the up- and down-regulated exon cassette, alternative last exon (ALE) and intron retention (IR) events in the *Sam68:Slm2^dko^* cortex. (D–F) Representative RT-PCR analysis of the splicing pattern of *Kif21a* exon 30 (**D**), *Arhgap12* exon 7a (**E**) and *Kans1l* exon 11 (**F**) during development of the wild-type cortex. (G–I) Representative RT-PCR analysis of the splicing pattern of *Kif21a* exon 30 (**G**), *Arhgap12* exon 7a (**H**) and *Kans1l* exon 11 (**I**) in the wild-type, *Sam68^ko^*, *Slm2^ko^* and *Sam68:Slm2^dko^* cortices at the indicated developmental stages. All graphs report the densitometric analysis of the PSI of the alternative exon and represent the mean ± SE of three independent samples. (D–I) Statistical analyses were performed with one-way ANOVA, Tukey's multiple comparisons test; *P< 0.05; **P< 0.01; ***P< 0.001; ****P< 0.0001.

The above data showed that the expression and splicing activity of Sam68 and SLM2 in the cortex are partially overlapping and these STAR proteins jointly control some shared target genes during cortical development, but with different strengths. Thus, we asked whether maintenance of a joint splicing control by separate Sam68 and SLM2 proteins is important for ensuring proper splicing of these target genes and for brain development. To address these questions, we generated a mouse model in which both Sam68 and SLM2 expression was ablated. *Sam68^−/−^* male mice are sterile (30) and females display strongly reduced fertility (29), whereas *Slm2^−/-^*mice are fertile (24). Hence, we first crossed *Sam68^+/−^* heterozygous mice with *Slm2^−/-^*mice to obtain the *Sam68^+/−^*:*Slm2^−/−^* genotype. These mice were then intercrossed in the attempt to increase the frequency of double knockout (*Sam68*:*Slm2^dko^*) mice (Supplementary Figure S4A). Strikingly, genotype analysis of newborn pups (P5) revealed a much lower frequency of dKO mice than expected (5.25% versus 25%), as we obtained only 21 dKO pups out of the 401 that were born from the mating of *Sam68^+/−^:Slm2^−/−^* mice (Supplementary Figure S4B). *Sam68^−/−^* mice display reduced perinatal viability, with 25–30% of newborn pups dying before weaning (25,29). To understand if the reduced frequency of dKO mice was due to fetal or perinatal lethality, we examined the genotype of embryos at the last day of gestation (E18.5). Analysis of 60 embryos from 8 different pregnancies showed the expected frequency of dKO mice (>25%; Supplementary Figure S4C), indicating that concomitant ablation of *Slm2* exacerbates the perinatal lethality of *Sam68^−/−^* mice. Furthermore, unlike the *Sam68^−/−^* mice (25,29), the few dKO mice that survived at birth displayed reduced viability (Supplementary Figure S4D). Western Blot analysis of extracts obtained from dKO cortices at the time of euthanasia confirmed the complete absence of both proteins in these mice (Supplementary Figure S4E). Importantly, the dynamic splicing pattern of *Nrxn1* and *Stxbp5l* was completely suppressed in the developing cortex of *Sam68*:*Slm2^dko^* mice (Figure 4A). Thus, concomitant ablation of these paralog RBPs strongly impairs mouse viability and disrupts the dynamic splicing of their common targets.

### Sam68 and SLM2 contribute to regulate the splicing program of the developing cortex

Our data showed that there is a dynamic regulatory cross-over between Sam68 and SLM2, but also some degree of redundancy that may reduce the amplitude of splicing changes caused by individual knockout at key points during cortical development. To evaluate more widely the impact of joint loss of Sam68 and SLM2 on the transcriptome of the embryonic cortex, we carried out RNA-sequencing (RNA-seq) analysis of polyadenylated (polyA+) RNA from the wild-type and dKO cortex at E18.5, before the lethal phenotype takes place. Bioinformatics analyses using the FAST database as reference (38,39) identified 293 splicing events that are differentially regulated in the dKO cortex compared to wild type (fold change ≥ 1.5; *P*-value < 0.05; Figure 4B). Exon cassette (EC; 17.7%) and alternative last exon (ALE; 14.7%) were the most regulated splicing patterns, followed by intron retention (IR; 7.8%). Furthermore, 54% of the regulated events are not annotated as alternative in the FAST database (Figure 4B), indicating that Sam68 and SLM2 are necessary for the regulation of exons and introns that were considered as constitutively spliced to date. Our analysis also showed that ECs were equally up- and downregulated in the dKO cortex, while ALE and IR events were prevalently downregulated (Figure 4C). Validation of 14 arbitrarily selected events confirmed the reliability of these bioinformatic analyses (Supplementary Figure S5A).

To identify targets of Sam68 and SLM2 that may be either specific to the cortex or dependent on their concomitant ablation, we compared the splicing events identified in the dKO cortex by RNA-seq (Supplementary Table S2) and RT-PCR analyses (Supplementary Figure S5B) with those previously reported as SLM2 targets in the hippocampus (20,21) or as Sam68 targets in NPCs, hippocampus and cerebellum (18,32,47). This analysis indicated that seven of the eight known targets of SLM2 were also differentially regulated in the dKO cortex. By contrast, many Sam68 targets found in other studies were not identified by our analysis (*n* = 704), which probably reflects the different brain regions and developmental stages analyzed. At the same time, we found 277 new events that are either specific for the dKO phenotype or the embryonic cortex (Supplementary Figure S6A; Supplementary Table S3).

Sam68 and SLM2 bind to bipartite motifs (named b1 and b2 sites in Supplementary Tables S4) comprising the (U/A)AA core sequence (48). However, these motifs are enriched in many introns. To search for features that are required for sensitivity to Sam68/SLM2 regulation, we computed the occurrence of the b1 and b2 motifs within 1kb upstream and downstream of six representative target exons (*Nrxn1-3*, *Stxbp5l*, *Kansl1l* and *Arhgap12*) in comparison to non-target exons of the same genes (Supplementary Table S4). For all these exons, Sam68 and SLM2 promote skipping. Our data show that, beside the *Nrxn2* AS4 exon that is highly specific for SLM2, all target exons display high b1 + b2 occurrence (column T in Supplementary Table S4). However, this feature is also observed at other non-target exons of the same genes. Specificity is likely achieved by occurrence of the first motif (b1) in proximity of the 3′ splice site of the skipped exons (column D in Supplementary Table S4). For instance, in the *Nrxn1-3* genes only the AS4 exon shows a high b1 + b2 score and a short distance (15–26 nt) of the first b1 motif from the 3′ splice site. By contrast, other exons with high b1 + b2 scores (i.e. exon 8 in *Nrxn1* and exons 6, 12 and 21 in *Nrxn3*) display the first b1 motif at > 70 nt from the 3′ splice site (Supplementary Table S4). A similar distribution was also observed in the other representative targets (Supplementary Figure S7; Supplementary Table S4). Thus, a combination of motif density and distance from the splice site is likely required to establish susceptibility to Sam68 and/or SLM2 control.

Next, we performed time-course analyses of select targets identified in the dKO cortex to test whether they are also developmentally regulated. We found that *Kif21a* exon 30 (Figure 4D), *Wink1* exon 25 and *Nrxn3* exon 21 (Supplementary Figure S6B,C) are dynamically regulated during development, similarly to what observed for the Sam68/SLM2 common targets (*Nrxn1* and *Stxbp5l*) and the SLM2-specific target *Nrxn2* (Figure 3A, B; Supplementary Figure S3A). By contrast, some other splicing events showed a steadier developmental pattern (*Arhgap12* exon 7a and *Kansl1l1* exon 11; Figure 4E, F). To test whether the different regulation of these exons was associated with dependency on Sam68, SLM2 or both, we evaluated their regulation in knockout mice. As expected from their common regulation by the two STAR proteins, skipping of the *Nrxn1* and *Stxbp5l* exons was further enhanced by combined knockout of *Sam68* and *Slm2* throughout cortical development, indicating their partially redundant role (Supplementary Figure S6D, E). Likewise, although splicing of *Kif21a* (Figure 4G) and *Wnk1* exons (Supplementary Figure S6F), was prevalently dependent on Sam68, ablation of *Slm2* contributed to its skipping at birth (P0), and concomitant knockout of both RBPs exerted a stronger effect than single knockouts. As a consequence, the dynamic pattern of these two exons was almost completely suppressed in the developing dKO cortex (Supplementary Figure S6G, H). Splicing of the developmentally-regulated *Nrxn3* exon was under specific control of SLM2 (Supplementary Figure S6I), whereas the non-dynamic exons in *Arhgap12* and *Kansl1l1* were only affected by *Sam68* ablation at all developmental stages analyzed and further knockout of *Slm2* only mildly enhanced this effect at P30 (Figure 4H,I). Interestingly, the only SLM2 target that was not differentially regulated in the dKO cortex at E18.5 (lysoPLD/ATX exon 25; Supplementary Figure S5B) is also modulated during development (Supplementary Figure S6J), concomitantly with the post-natal increase in SLM2 expression. Moreover, splicing of this exon was affected in the adult dKO cortex (Supplementary Figure S6K). Thus, dynamic splicing regulation of Sam68/SLM2 target exons during cortical development is associated with either partial or complete dependency on SLM2 and is likely dictated by the altered balance of the expression of these paralog RBPs after birth.

### Combined ablation of Sam68 and SLM2 expression impairs neurogenesis and neuronal maturation

**Figure 5.**
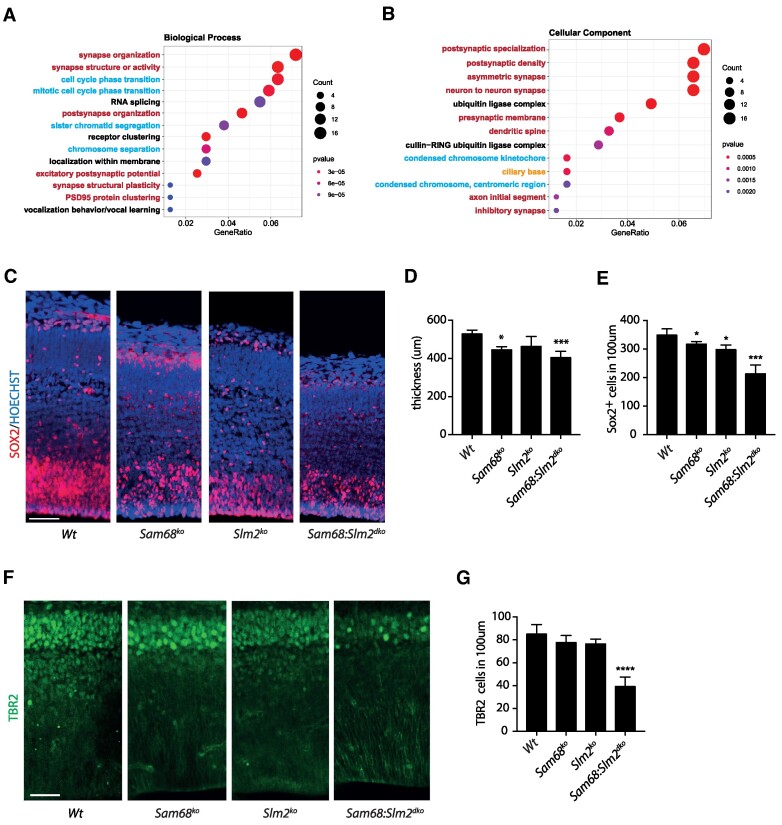
Combined ablation of Sam68 and SLM2 expression impairs neurogenesis. (**A**, **B**) Gene Ontology (GO) analysis of the genes regulated at splicing level in the *Sam68:Slm2^dko^* cortex. Dots’ size in the graph is proportional to the number of genes in each category; dots’ color represents the enrichment grade, with red indicating high enrichment score and blue indicating low enrichment score. Gene Ratio indicates the number of the differentially regulated genes on the total of genes in the GO term. P-value was calculated with Classic Fisher's Exact test. (C–E) Representative images (**C**) and quantitative analysis of the cortical thickness from ventricle to pial surface (**D**) or of neuronal cells stained for SOX2 (**E**) in the cortex of wild-type, *Sam68^ko^*, *Slm2^ko^* and *Sam68:Slm2^dko^* embryos at E18.5. (F, G) Representative images (**F**) and quantitative analysis (**G**) of immunofluorescence for the neural progenitor marker TBR2 in the cortex of wild-type, *Sam68^ko^*, *Slm2^ko^* and *Sam68:Slm2^dko^* embryos at E18.5. Histograms show the data (mean ± SE) of three independent male embryos for each genotype used for the analyses. Statistical analyses were performed using the one-way ANOVA test; *P< 0.05; ***P< 0.001; ****P< 0.0001. Scale bar = 50 μm in (C) and 33 μm in (F).

Functional annotation of the genes regulated at splicing level in the *Sam68*:*Slm2^dko^* cortex highlighted pathways of direct relevance for brain development and function. In particular, terms related to cell cycle progression and to synapse organization and function were significantly enriched (Figure 5A,B). Terms related to neuron adhesion and synaptic structure/function were also enriched among the 51 genes overlapping with studies on single knockout mice (Supplementary Figure S8A, Supplementary Table S3). Cell cycle control is crucial for neurogenesis during cortical development. STRING analysis of splicing-regulated genes highlighted a network of proteins involved in spindle orientation and/or dynamics (Supplementary Figure S8B), a mitotic step that is critical for proper cortical development (49). Accordingly, histological analysis of Hoechst-stained sections indicated that cortical thickness was significantly reduced at E15.5 (Supplementary Figure S8E,F) and at the end of fetal neurogenesis in the *Sam68*:*Slm2^dko^* embryos, and, to a lesser extent, in *Sam68^−/−^* mice at E18.5 (Figure 5C, D). To test whether this defect was associated with altered neurogenesis, we analyzed NPC markers in the developing cortex. Immunofluorescence analysis of the stemness marker SOX2 indicated a reduced number of NPCs at E15.5 and 18.5 in the *Sam68^−/−^* and *Slm2^−/−^* cortex, with a stronger depletion of these proliferation-proficient cells in the dKO (Figure 5C,E; Supplementary Figure S8E,G). Reduced NPC proliferation was further confirmed by staining with the proliferation marker Ki67 at E15.5 (Supplementary Figure S8E, H). Strikingly, transient-amplifying neural progenitors, which are marked by the transcription factor TBR2 (50), were only significantly reduced in the dKO mice (Figure 5F, G).

**Figure 6.**
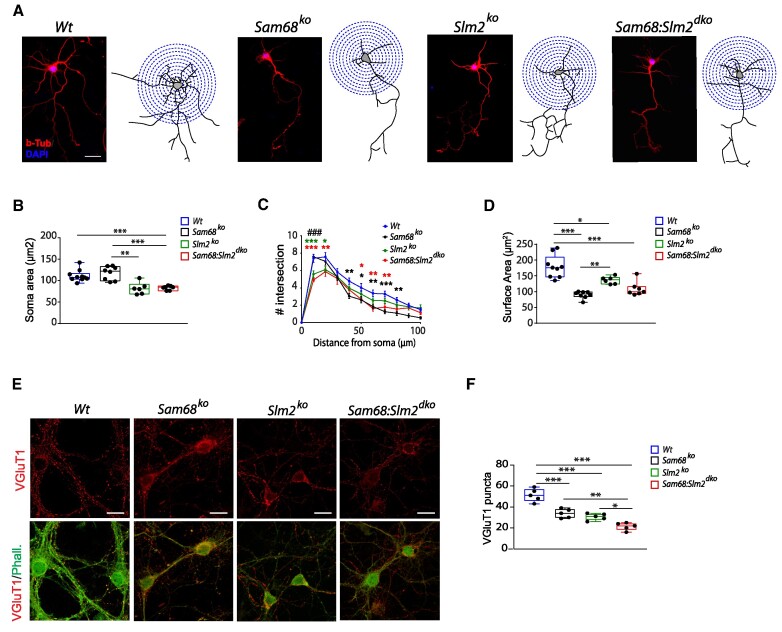
Combined ablation of Sam68 and SLM2 expression impairs neuronal maturation, dendritic arborization and synaptic organization of cortical neurons. (**A**) Representative images of cortical neurons of the indicated genotypes showing immunostaining with β-Tubulin and DAPI and the relative 3D-reconstructed structures (scale bar = 20 μm). (B–D) Box-and-whisker plots of soma area (**B**) and overall surface area (**D**) and graph of the distribution of the intersections at different distances from the soma (**C**) of individual cortical neurons of the indicated genotypes (7–10 neurons per culture were randomly selected, n = 4 mice for each experimental group). (**E**) Representative images of immunofluorescence for VGluT1 (red) and phalloidin (green) in cortical neurons of the indicated genotypes. (**F**) Box-and-whisker plots of the number of VGluT1 puncta (in an area of 400 μm^2^) on phalloidin positive neurons of the indicated genotypes (*n* = 5 mice for experimental group). In the box-and-whisker plots, the center line shows the median value, edges are upper and lower quartiles, whiskers show minimum and maximum values, and each point is an individual set of experiments. Data were analyzed by one-way ANOVA followed by a Bonferroni *post hoc* test, *P< 0.05, **P< 0.01, ***P< 0.001.

Splicing-regulated genes were also related to properties of the differentiated neurons, such as synapse structure and function (Figure 5A, B). Moreover, STRING analysis highlighted a network of interconnected synaptic proteins encoded by genes (*Nrxn1-3*, *Cask*, *Shank3*; Supplementary Figure S8C, D) whose mutations have been associated with neurodevelopmental defects and/or syndromes (15,51–53). Thus, we analyzed neuronal morphology of cortical neurons isolated from the E14.5 cortex of wild-type, *Sam68^−/−^*, *Slm2^−/−^* and dKO mice and cultured *in vitro* for 5 days (5 DIV). The morphology, complexity and branching of the dendrites and the size of the soma were evaluated by Sholl's analysis in βIII-tubulin positive neurons (54). *Sam68*:*Slm2^dko^* neurons displayed lower complexity and ramification compared to wild-type neurons, comprising a reduced soma area (Figure 6A, B) fewer branched processes and an overall reduced number of intersections with the concentric circles (Figure 6C), particularly at distances ranging between 10 and 80 μm from the soma center (Figure 6A, C). Moreover, *Sam68*:*Slm2^dko^* neurons also showed a lower ramification area of the neurites (Figure 6D). Interestingly, these defects were differentially associated with the two paralog RBPs. *Sam68^−/−^* neurons presented a conserved soma area (Figure 6B) but featured a reduced ramification of neurites and lower number of intersections mainly at distal portions (40–80 μm) from the soma (Figure 6C). These defects likely contributed to the reduced surface area observed in the *Sam68*:*Slm2^dko^* neurons (Figure 6D). Conversely, ablation of *Slm2* likely accounted for the reduced size of the soma and the fewer branched processes in the proximal portions (10–20 μm) from the soma (Figure 6B, C).

Since formation of dendritic arborization is related to the level and the distribution of the synaptic inputs received by the neuron (55), we analyzed the localization of VGluT1, an excitatory pre-synaptic marker, in cortical neurons counterstained with phalloidin. Analysis of the co-localization between VGLuT1 and phalloidin signals showed a decrease in presynaptic puncta, on both neurites and soma of cortical neurons of all genetic backgrounds with respect to wild-type neurons (Figure 6E, F). Furthermore, dKO neurons displayed significantly fewer VGLuT1 puncta than *Sam68^−/−^*and *Slm2^−/−^* neurons (Figure 6F), indicating that both RBPs are required for proper assembly of presynaptic terminals and that their concomitant ablation further impairs neuronal connections.

Collectively, these results indicate that Sam68 and SLM2 contribute to both overlapping and unique processes during cortical development, including neurogenesis, neuronal maturation, dendritic arborization and synaptic organization.

### Dual ablation of Sam68 and Slm2 causes hydrocephalus *in vivo*

Histological analysis of the brain in the few surviving *Sam68*:*Slm2^dko^* mice (*n* = 4), followed by quantitative analyses, showed the presence of highly dilated cerebral ventricles with respect to wild-type mice (Supplementary Figure S9A, B), suggestive of a hydrocephalus. Moreover, several genes (*n* = 17) whose splicing is altered in the *Sam68*:*Slm2^dko^* brain were shown to be mutated in patients affected by hydrocephalus or to cause a hydrocephalus phenotype in mice (56) (Supplementary Figure S9C, Supplementary Table S5). Thus, since the presence of a hydrocephalus can represent a cause of increased perinatal lethality, we analyzed E18.5 embryos to identify if there are corresponding early predictive changes in embryonic head and brain morphology before the lethal phenotype is manifested.

**Figure 7.**
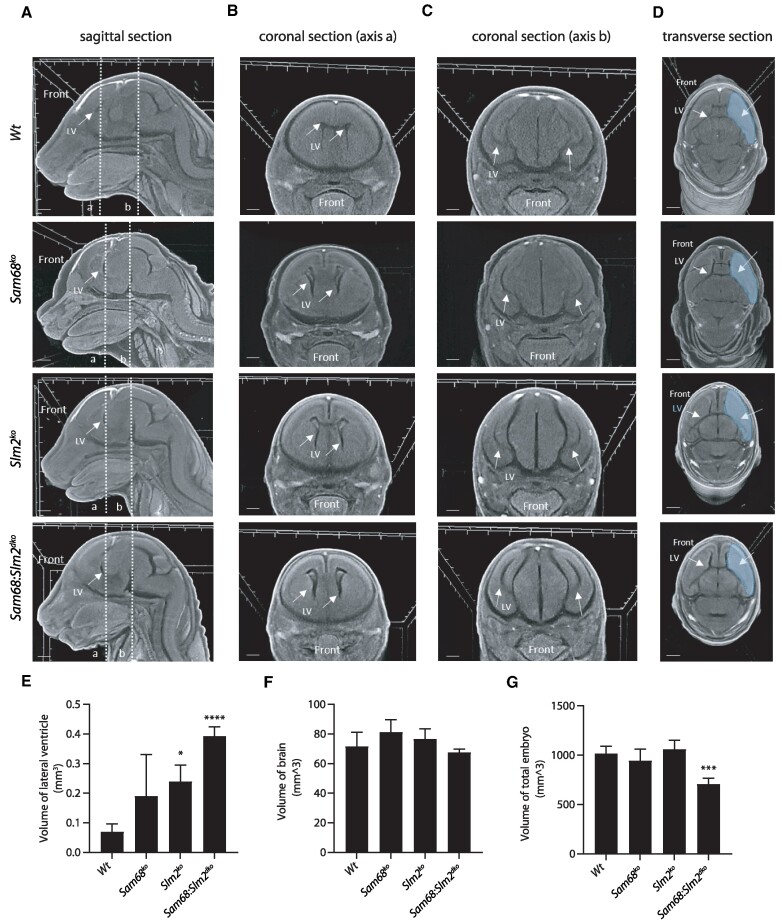
3D-MicroCT virtual histology of embryo brains. (A–D) CT imaging of the head of E18.5 embryos of the indicated genotypes acquired along a virtual median sagittal plane (**A**), two coronal planes (**B, C**) sectioned according to the a and b axes defined in (A), and a transverse plane (**D**). Arrows indicate the lateral ventricles; the hatched area in (D) indicates the cortical region in a central cross section (scale bar = 1 mm). (E–G) The bar graphs indicate the volume of lateral ventricle (**E**), the volume of brain (**F**) and the volume of the total embryo (**G**); *n* = 4 mice for each experimental group. Data were analyzed by one-way ANOVA followed by a Bonferroni *post hoc* test,, *P< 0.05, ***P< 0.001, ****P< 0.0001.

To evaluate the three-dimensional conformation of the embryonic brain, we performed X-Ray Micro-Computed Tomographic analysis (MicroCT). Each embryo was scanned along the sagittal plane, two separate coronal planes and the transverse plane to quantify the volumetric parameters of the lateral ventricles (Figure 7A–D). This analysis highlighted a significant increase in the volume of the lateral ventricles in *Sam68*:*Slm2^dko^* brain (*n* = 6; Figure 7E). This defect was also observed to a lesser extent in the single knockout brains, indicating an additive effect of concomitant *Sam68* and *Slm2* ablation. Interestingly, while *Sam68*:*Slm2^dko^* embryos were significantly smaller than wild-type or single knockout embryos at E18.5, the overall volume of their brain was not reduced (Figure 7F, G), suggesting that it was compensated by the enlargement of the ventricles. These results indicate that increased lethality of *Sam68*:*Slm2^dko^* mice is associated with enhanced defects of cerebral development during fetal life, and that it has been evolutionary necessary to maintain separate Sam68 and SLM2 proteins to enable proper brain development and survival.

## Discussion

The production of specific splice variants at proper times in the developing brain ensures fine-tuning of key processes, such as neurogenesis (32), synapse specification (18,57,58) and synaptic plasticity (16–18,58,59). The brain splicing program is dictated by the expression of both CNS-restricted and ubiquitous splicing factors. Herein, we found that the differential expression of two highly homologous paralog splicing factors, the ubiquitous Sam68 and the CNS/testis-specific SLM2, is required for proper neuron maturation and brain development. Previous studies indicated that Sam68 and SLM2 are co-expressed in the mouse cortex (26) and that they affect both unique and common splicing events (18,24,36). Herein, by generating a double knockout mouse model and by comparing its phenotype with that of single *Sam68* and *Slm2* knockouts we found that Slm2/Sam68-dependent splicing control is dynamically regulated during cortical development, and that these highly homologous RBPs interact to dynamically control splicing patterns of key genes required for cortical development and function. Moreover, molecular and developmental analyses of these mice show that Sam68 and SLM2 have both redundant and individual roles during pre- and postnatal cortical development. Characterization of the *Sam68*:*Slm2^dko^* mouse model uncovered phenotypes caused by joint ablation of these two RBPs, such as hydrocephalus development, indicating that maintenance of separate Sam68 and SLM2 proteins is critical for survival. Interestingly, more subtle versions of these phenotypes can also be detected within the *Sam68* and *Slm2* single knockouts, but they were previously overlooked because they manifest at a less extreme level.

By carrying out a developmental time course analysis, we show that Sam68 expression is high during neurogenesis and it steadily declines after birth, whereas SLM2 displays an inverse pattern of expression. Indeed, SLM2 expression is restricted to the cortical plate in the embryonic cortex and steadily increases to become uniformly distributed in the adult cortex. Nevertheless, despite these temporal expression gradients during cortical development, both proteins are co-expressed in most mature cortical neurons. Importantly, while SLM2 expression is independent of Sam68 activity, Sam68 expression levels are modulated by SLM2 at postnatal stages, indicating a hierarchical cross-regulation between these homologous proteins during cortical development.

Sam68 and SLM2 were shown to self-control their expression by promoting the inclusion of non-coding exons downstream of the canonical stop codon, thus eliciting NMD-mediated degradation of their own transcript (21). However, these studies were all performed in adult mice. Herein, analysis of single knockout mice during cortical development show that this feedback mechanism is developmentally relevant for physiological tuning of the expression and activity of these paralog RBPs. Moreover, we show that the extent of this autoregulation is dictated by their relative expression levels, being maximal when they are expressed at high levels and minimal when their expression is lower. The autoregulation of Sam68 and SLM2 might be required to limit the potentially negative effects of their uncontrolled expression. Indeed, up-regulation of Sam68 (45) and SLM2 (46,60) is associated with tumorigenesis. In many cases, the oncogenic functions of Sam68 (61–65) and SLM2 (60) have been attributed to their direct regulation of RNA processing events in genes that control cell proliferation and survival. On the other hand, excessive expression of Sam68 triggers cell death (66). Thus, autoregulation of Sam68 and SLM2 in normal cells is likely required to ensure the physiological function of these RBPs, while preventing the onset of pathological conditions.

SLM2 was shown to induce NMD-mediated control of the third paralog gene: *Slm1/Khdrbs2*. SLM2 represses expression of SLM1 in the CA region of the hippocampus, restricting it to the dentate gyrus where SLM2 is absent (27). We now document that SLM2 also regulates the expression of Sam68 in the cortex by NMD. However, our developmental analysis indicates that this repression is exerted only at late stages of post-natal development, when SLM2 expression is maximal. Moreover, the effect of SLM2 on Sam68 expression is not fully penetrant, differently from that exerted on SLM1 (27). This is possibly due to the high expression levels of Sam68, which is a ubiquitous and highly expressed protein, and/or to lower affinity of SLM2 for the Sam68 transcript with respect to the SLM1 transcript. Taken together, these observations suggest that, when its concentration rises above a certain threshold, SLM2 represses the other paralog STAR proteins, thus acting as a hierarchical player in establishing their relative expression levels. Thus, these findings uncover a developmental switch between paralog RBPs that is mediated by the SLM2-dependent NMD mechanism.

Interestingly, except for the *Nrxn2* AS4 exon (24), SLM2 does not appear to have unique splicing targets (20,21,36,58). Nevertheless, the relative efficacy of Sam68 and SLM2 for their common targets varies during development. For instance, while both RBPs repress inclusion of *Nrxn1* exon 20, SLM2 is much more efficient than Sam68 in the P30 cortex. In comparison, when Sam68 is expressed at higher levels than SLM2 (e.g. E15.5), this exon is included in more than 50% of the *Nrxn1* transcripts, whereas it becomes prevalently skipped (>70%) at P30, when SLM2 is the predominant paralog RBP in the cortex. Thus, although SLM2 appears to have a limited number of splicing targets, its developmental-regulated expression pattern ensures a dynamic regulation of joint target exons that are also susceptible to its activity. On the other hand, exons that are mainly controlled by Sam68 display a steady splicing pattern during cortical development. In this way, two highly homologous paralogs may have evolved to allow differential regulation of partially overlapping subsets of neuronal genes during cortical development. Furthermore, acquisition of a paralog gene establishes a mechanism to partially compensate for the loss of one of these RBPs. Indeed, while ablation of SLM2 is compatible with life and that of Sam68 only partially reduces viability (24,25), concomitant knockout of both genes was highly detrimental, with very few animals surviving perinatally and displaying a strong phenotype. This helps explain why despite having overlapping functional activity distinct Sam68 and Slm2 genes have been maintained since their divergence from a common ancestor gene 520–610 million years ago.

Functional annotation of the genes regulated at splicing level in the *Sam68*:*Slm2^dko^* cortex highlighted terms related to the synapse, neuron morphology, cell cycle and mitotic spindle regulation. STRING analysis also highlighted an interacting synaptic network involving genes (i.e. *Nrxn1-3, Cask and Shank3*) whose mutations have been associated with neurodevelopmental defects and/or syndromes (15,51–53). The phenotypes identified in *Sam68*:*Slm2^dko^* mice are compatible with defects in these structures and functional processes. Indeed, we detected significant defects in soma size and in neurite arborization of the dKO cortical neurons differentiated *in vitro*. Interestingly, these defects were the sum observed in single knockouts, indicating that Sam68 and SLM2 contribute to different aspects of neuron maturation. Likewise, impairment of neurogenesis was more dramatic in the dKO cortex, with stronger reduction of SOX2-positive neural progenitor cells and of cortical thickness compared to single knockouts. Moreover, a significant reduction of TBR2-positive cells, a subset of transient-amplifying progenitors that are already committed to differentiate into neurons (50), was only detected in the dKO cortex. Another peculiar feature observed in *Sam68*:*Slm2^dko^* mice was the development of a frank hydrocephalus. The few surviving mice died within 30 days from birth and displayed great expansion of the cerebral ventricles. This defect was already detected at the end of fetal development by computed tomography and likely contributed to perinatal lethality. Hydrocephalus is often caused by an impairment in ciliated ependymal cells, and ciliary base was one of the terms enriched among the splicing-regulated genes in the cortex of *Sam68*:*Slm2^dko^*mice. Thus, double knockout of these paralog genes enabled defects, such as hydrocephalus and neuronal connectivity, to be more clearly distinguished with respect to the single knockouts, by either removing redundancy or a more precise developmental control.

Interestingly, other paralog RBPs undergo developmental regulation in a similar manner with functional consequences for brain development. For instance, the ubiquitous PTBP1 was shown to control neuron-restricted PTBP2 expression by an NMD-associated splicing event (22). Also in this case, several developmental-regulated exons were shown to be differentially sensitive to PTBP1 and PTBP2 (67), even though the mechanism involved in such specificity was not investigated. However, PTBP2 also has a distinct function in dictating the timing of axonogenesis during cortical development, arguing against the complete functional redundancy between these paralog RBPs (68). Thus, the PTBP paralogs switch not only fine-tunes a redundant splicing program, but it also exerts effects on specific aspects of neuronal biology. Likewise, herein we show that up-regulation of SLM2 in the postnatal cortex allows enhanced expression of synaptic isoforms, such as the NRXN1-3 AS4-skipped splice variants, which are associated with specific cognitive functions (14,15,20,21). Thus, cross-control between paralog RBPs may allow a more sophisticated regulation of specific splicing events in the developing cortex and/or in response to neuronal stimulation (16–18). At the same time, co-expression of Sam68 and SLM2 in mature neurons may establish a redundant mechanism of splicing control that is essential to ensure the correct structural and functional formation of the cortex.

## Supplementary Material

gkae071_Supplemental_Files

## Data Availability

All data generated or analyzed during this study are included in this article. The RNA sequencing data are available in Gene Expression Omnibus under accession number GSE229749.
