# Supplementary material for: Differential expression of paralog RNA binding proteins establishes a dynamic splicing program required for normal cerebral cortex development
Source: Nucleic Acids Res. 2024 Feb 7;52(8):4167–84. doi: 10.1093/nar/gkae071 (PMC11077083; doi:10.1093/nar/gkae071)
Supplement: gkae071_Supplemental_Files [file gkae071_supplemental_files.zip › Supp_Fig_1_NEW_merged_compressed.pdf]

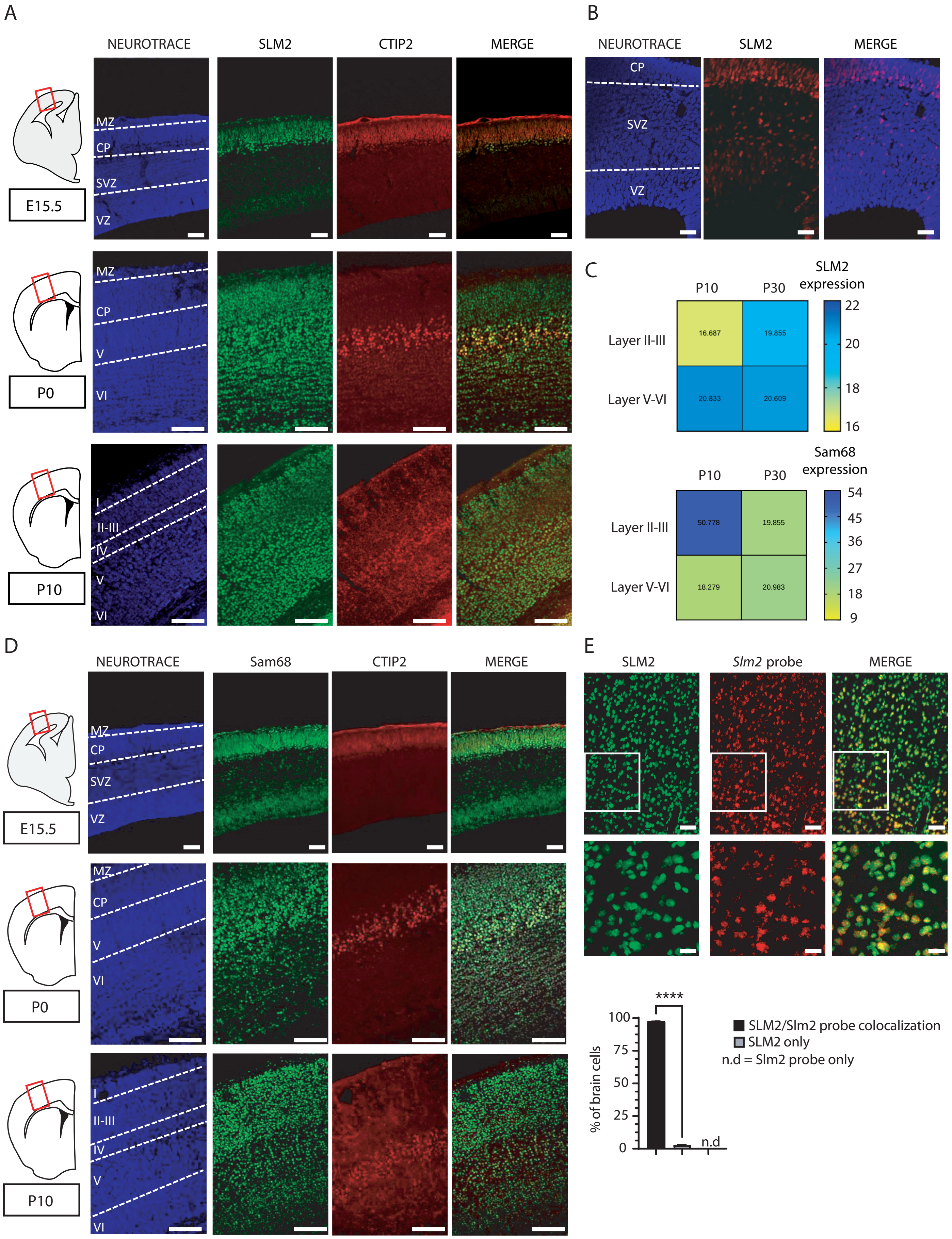

Supp. Figure 1

Supplementary Figure 1. Sam68 and SLM2 immunostaining in the developing mouse cerebral cortex. A) Confocal images of SLM2 (green), CTIP2 (red) and Neurotrace (blue) showing the differential distribution of SLM2 positive cells during development (from E15.5 to P10). The cortical regions corresponding to layers I-VI are marked by dotted lines in the Neurotrace image. A scheme of the cortical region examined is shown on the left of the panels. Scale bar 50µm. **B**) Confocal images of SLM2(red) and Neurotrace (blu) staining in the E15.5 mouse cortex showing the presence of SLM2 positive cells in both in CP and SVZ. Scale bar 50 µm. **C)** SLM2 (upper panel) and Sam68 (lower panel) heat map showing the expression gradient of the two proteins at P10 and P30 in the indicated layers of the cerebral cortex. **D)** Confocal images of Sam68 (green), CTIP2 (red) and Neurotrace (blue) showing the differential distribution of Sam68 positive cells during development (from E15.5 to P10). The cortical regions corresponding to layers I-VI are marked by dotted lines in the Neurotrace image. A scheme of the cortical region examined is shown on the left of the panels. Scale bar 50µm. **E)** Confocal images of SLM2 and *Slm2* RNA probe and relative bar graph that represents the percentage of SLM2 and Slm2 RNA probe colocalization and the single stainings in the layer V of the sensory motor cortex. Scale bar 50 µm (upper panel) and 15 µm (lower panel). Data represent the mean + standard error (SE) of at least three independent samples. Statistical analysis was performed independently for SLM2 and Slm2 probe expression by one way ANOVA Tukey's multiple comparisons test; ****P=0.0001.

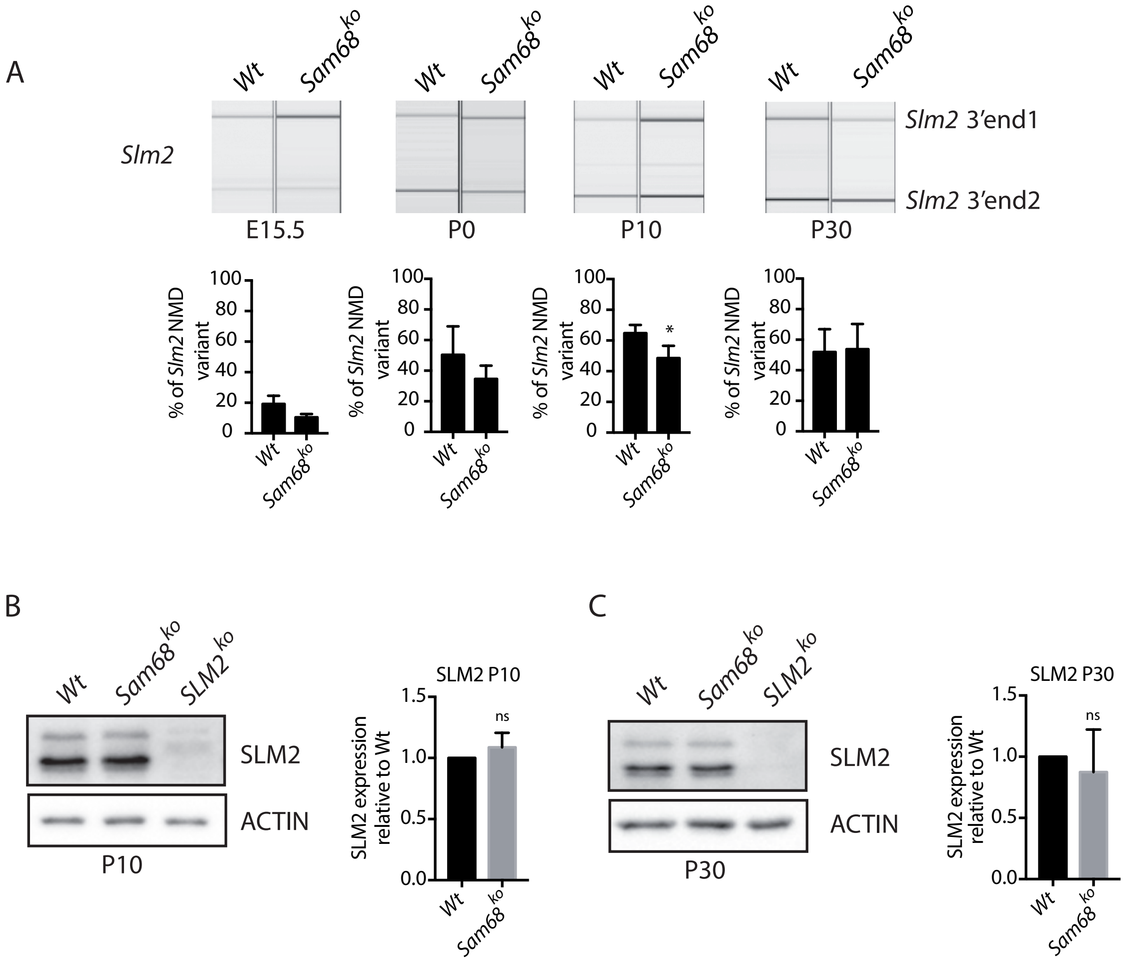

Supp. Figure 2

Supplementary Figure 2. NMD-mediated regulation of Sam68 and SLM2. A) Quantitative capillary RT-PCR analyses of the percentage of NMD-targeted alternative end 2 mRNA variant of *Slm2* in the *wild-type* and *Sam68 ko* cortex at the indicated developmental stages. Data are expressed as alternative end 2/alternative end 1+alternative end 2 and represent the mean + SE of at least three independent samples. Statistical analysis was performed by one-way ANOVA with respect to the E15.5 ratio for each gene; *P=0.05. **B,C)** Representative Western blot analysis of SLM2 protein expression in the *wild-type* and *Sam68 ko* cortex at P10 (**B**) and P30 (**C**). Bar graphs show the densitometric analysis of three independent samples. Statistical analysis was performed by Student's t-test; n.s.= not significant.

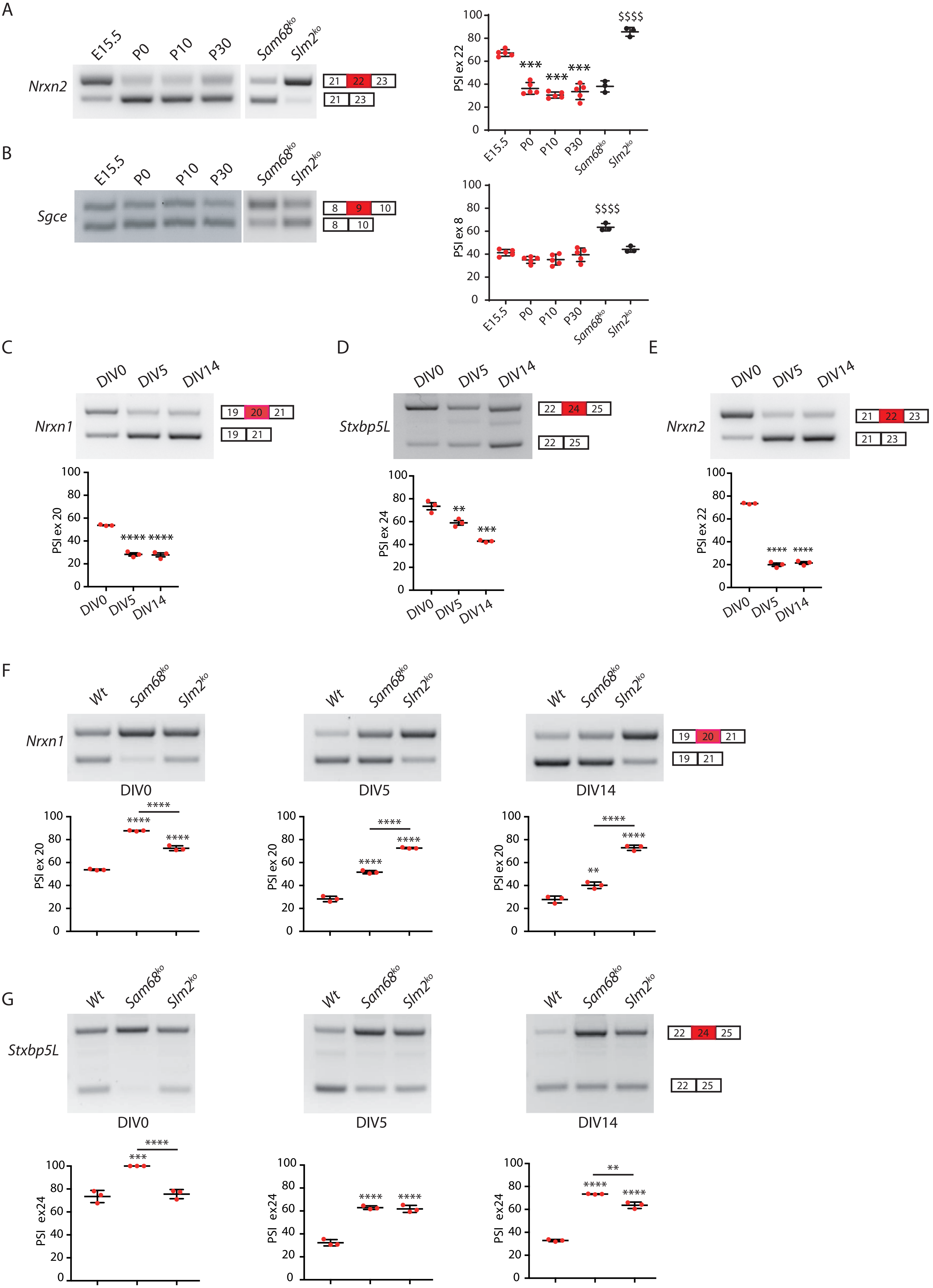
A
E15.5
P0
P10
P30
Sam68ko
Slm2ko
Nrxn2
21 22 23
21 23
PSI ex 22
***
$$$$
B
Sgce
8 9 10
8 10
PSI ex 8
C
DIV0
DIV5
DIV14
Nrxn1
19 20 21
19 21
PSI ex 20
****
D
Stxbp5L
22 24 25
22 25
PSI ex 24
**
E
PSI ex 22
F
Wt
DIV0
DIV5
DIV14
G
PSI ex24

Supplementary Figure 3. Developmental splicing regulation of Sam68- and SLM2-target genes. A,B) Representative RT-PCR analysis of the splicing pattern of *Nrxn2* exon 22 **(A)** and *Sgce* exon 9 **(B)** during development of the wild-type cortex and of $Sam68^{ko}$ and $Slm2^{ko}$ cortex at P30. **C-E)** Representative RT-PCR analysis of the splicing pattern of *Nrxn1* exon 20 **(C)**, *Stxbp5l* exon 24 **(D)** and *Nrxn2* exon 22 **(E)** during cortical neuron differentiation *in vitro* for 0, 5 and 14 days (days *in vitro*, DIV). **F,G)** Representative RT-PCR analysis of the splicing pattern of *Nrxn1* exon 20 **(F)**, *Stxbp5l* exon 24 **(G)** in wild-type, $Sam68^{ko}$ and $Slm2^{ko}$ cortical neurons and differentiated *in vitro* for 0, 5 and 14 days. **A-G)** All graphs show the PSI levels from densitometric analyses of the RT-PCR data (mean ± SE, n=4). Statistical analyses were performed by one-way ANOVA, Tukey's multiple comparisons test; *P<0.05, **P<0.01, ***P<0.001, ****P<0.0001.

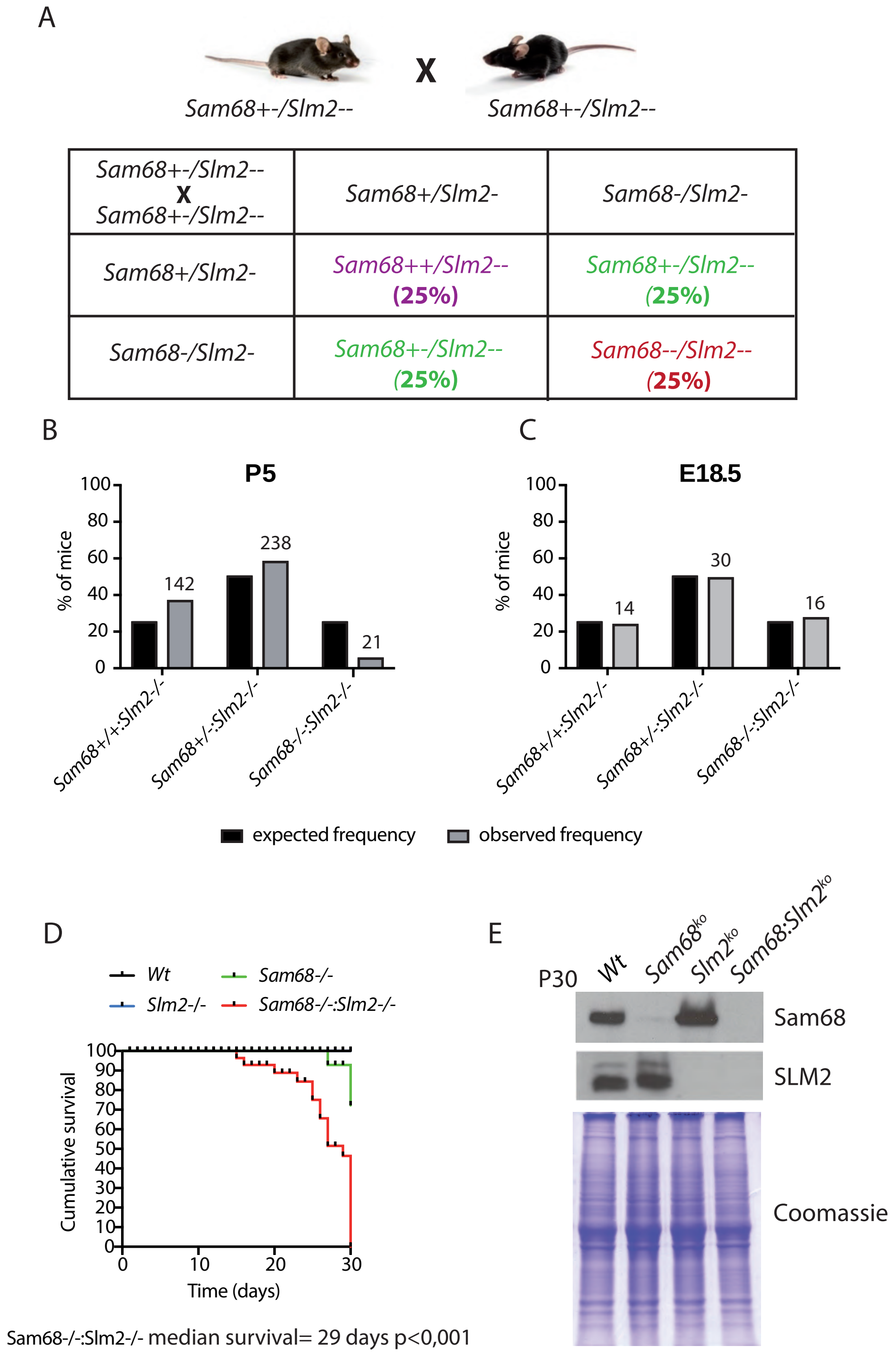

Supp. Figure 4

Supplementary Figure 4. *Sam68:Slm2dko* mice display a perinatal lethal phenotype. A) Scheme of the breeding assay used to generate *Sam68:Slm2dko* mice and the expected percentage of pups for each genotype. **B,C)** Bar graphs showing expected frequency (black) and the observed frequency (grey) at postnatal day (P) 5 (**B**) or at the last day of gestation (E18.5) (**C**). The number of pups obtained for each genotype is written on top of the grey bars. **D)** Kaplan-Meier survival curves of *wild-type* (*Wt*; n=30), *Sam68ko*(n=30) , *Slm2ko* (n=30) and *Sam68:Slm2-dko* (n=21). Statistical analysis was performed by the log-rank test (P<0.0001). **E)** Western blot analysis of P30 cortices from *wild-type* (*Wt*), *Sam68ko, Slm2ko* and *Sam68:Slm2dko* mice to confirm the absence of Sam68 and SLM2 in the dKO mice. Coomassie staining was used as loading control.

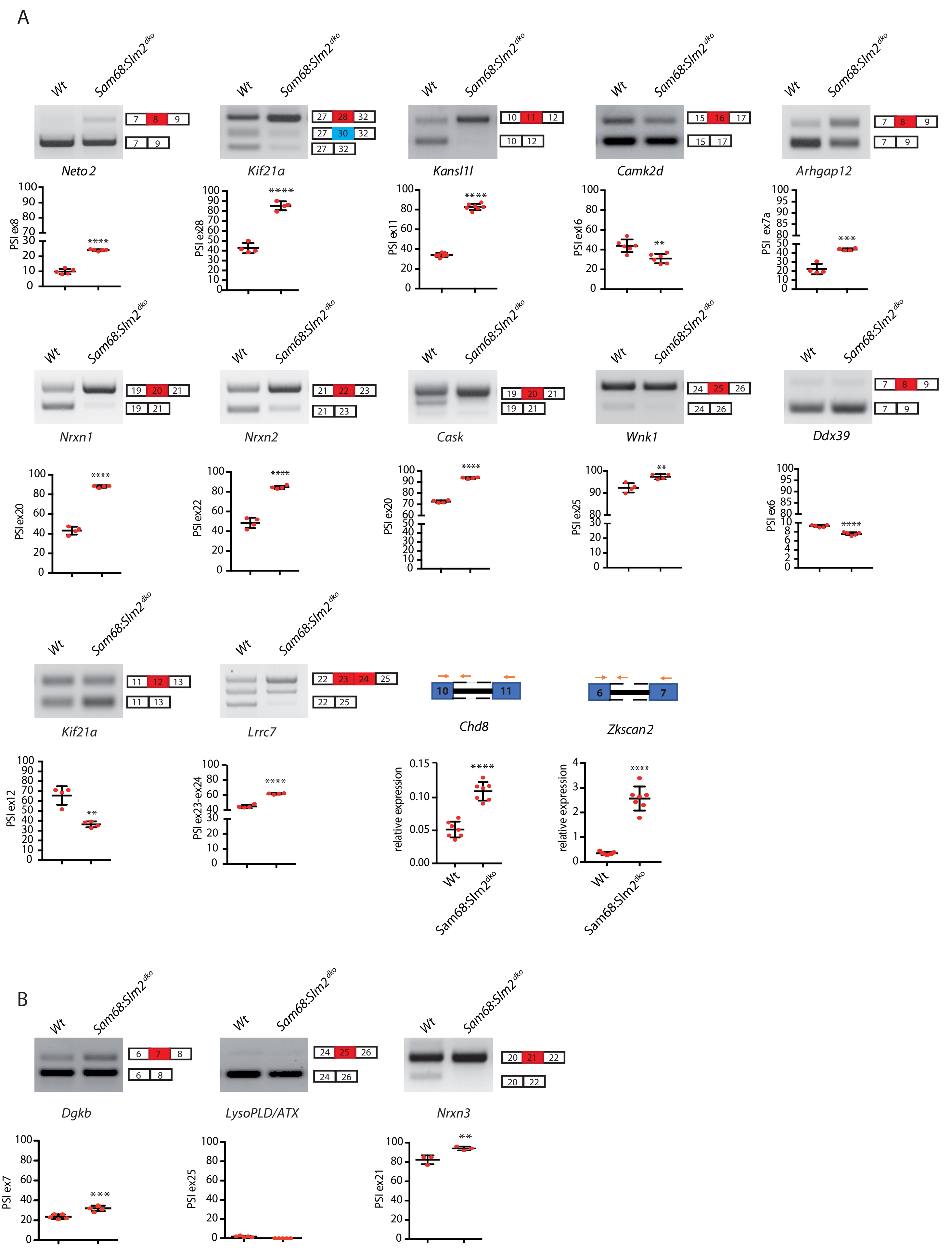

Supp. Figure 5

Supplementary Figure 5. Sam68 and SLM2 exert a widespread effect on splicing regulation in the developing cortex. A) Representative RT-PCR analysis of the indicated genes (n=14) used to validate the splicing analysis from the RNA-seq data. **B)** Representative RT-PCR analysis of the three known target genes of Slm2 that are regulated (Dgkb and Nrxn3) or not (LysoPLD/ATX) in the *Sam68:Slm2*^{dko} cortex. (**A-B**) All graphs show the PSI levels calculated by densitometric analysis of the RT-PCR data (mean ± SE, n=7 in A and at least n=3 in B). Statistical analysis was performed by Student's t-test; **P< 0.01, ***P<0.001, ****P<0.0001.

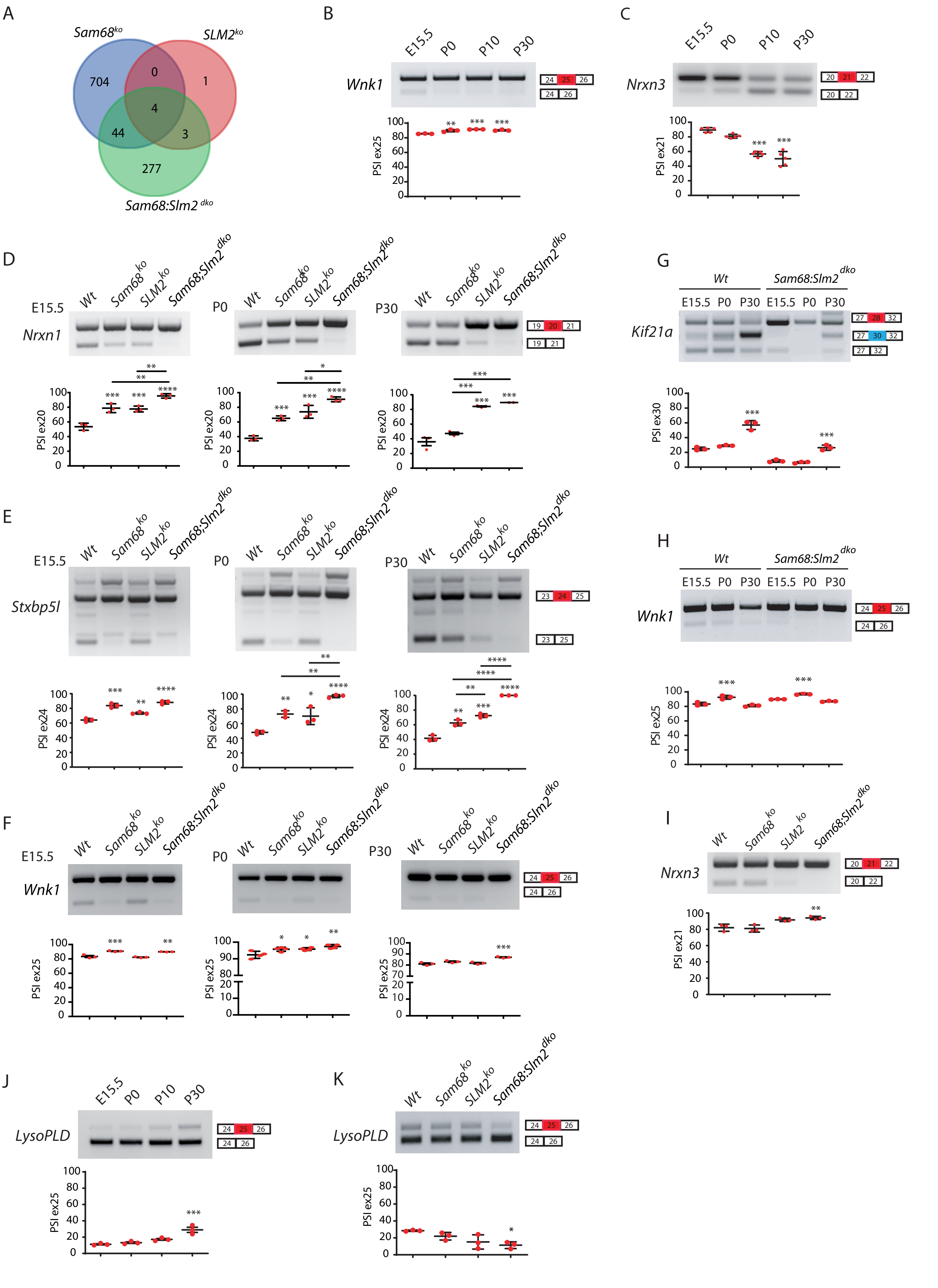

Supp. Figure 6

Supplementary Figure 6. Developmental control of splicing by Sam68 and SLM2. A) Venn diagram showing the overlap between splicing events differentially regulated in the *Sam68*^{ko} cortex, cerebellum and hyppocampus*, Slm2*^{ko} *hippocampus and Sam68:Slm2*^{dko} cortex. **B-C)** Representative RT-PCR analysis of the splicing pattern of *Wink1* exon 25 (**B**) or *Nrxn3* exon 21 (**C**) during development of the wild-type cortex. **D-F)** Representative RT-PCR analysis of the splicing pattern of *Nrxn1* exon 20 **(D)**, *Stxbp5l* exon 24 **(E)** and Wnk1 exon 25 **(F)** in the wild-type (*Wt*), *Sam68*^{ko}, *Slm2*^{ko} and *Sam68:Slm2*^{dko} cortex at the indicated age of development. **(G-H)** Representative RT-PCR analysis of the splicing pattern of *Kif21a* exon 30 (**G**) and Wink1 exon 25 (**H**) during development of the wild-type and *Sam68:Slm2*^{dko} cortex. **I)** Representative RT-PCR analysis of the splicing pattern of *Nrxn3* exon 21 in the wild-type (*Wt*), *Sam68*^{ko}, *Slm2*^{ko} and *Sam68:Slm2*^{dko} cortex at P0. **J,K)** Representative RT-PCR analysis of the splicing pattern of *LysoPLD* exon 25 during development of the wild-type cortex (**L**) and in the wild-type (*Wt*), *Sam68*^{ko}, *Slm2*^{ko} and *Sam68:Slm2*^{dko} cortex (**K**). (**B-K**) All graphs show the PSI calculated by densitometric analysis of the RT-PCR data (mean ± SE, n=3). Statistical analyses were performed with one-way ANOVA, Tukey's multiple comparisons test; *P<0.05; **P< 0.01; ***P<0.001; ****P<0.0001.

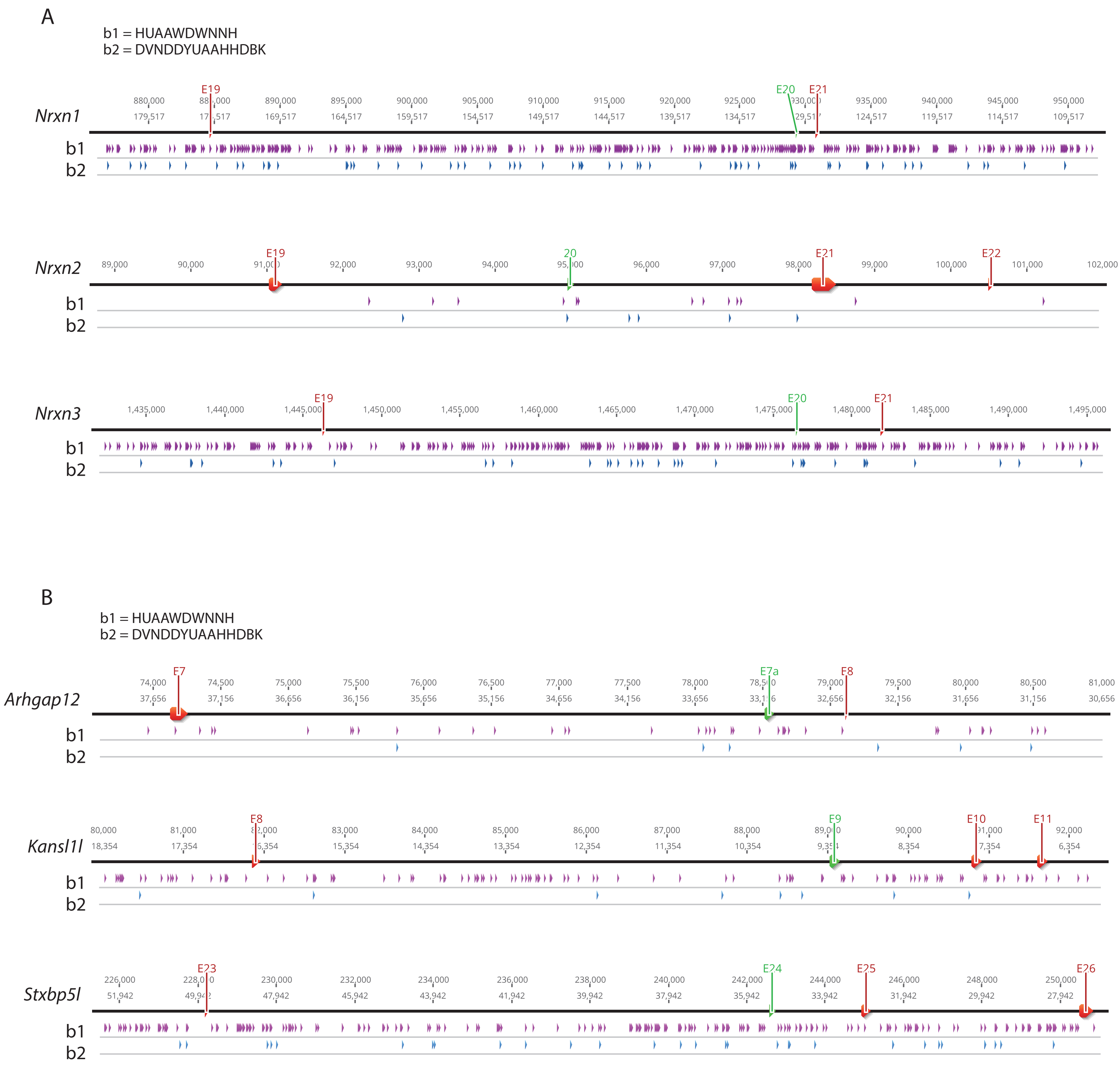

Supp. Figure 7

Supplementary Figure 7. Binding sites of Sam68 and SLM2 target genes. A,B) Representative images of Sam68/SLM2 binding site 1 (b1: HUAAWDWNNH; arrow head in purple) or Sam68/SLM2 binding site 2 (b2: DVND-DYUAAHHDBK; arrow head in blue) flanking (1kb regions 5'upstream and 3'downstream) the alternative spliced exons of *Nrxn1-3* (A) and *Arhagap12*, *Kansl1l* and *Stxbp5l* (B). The regulated exon for each gene is indicated in green, while the flanking exons are in red.

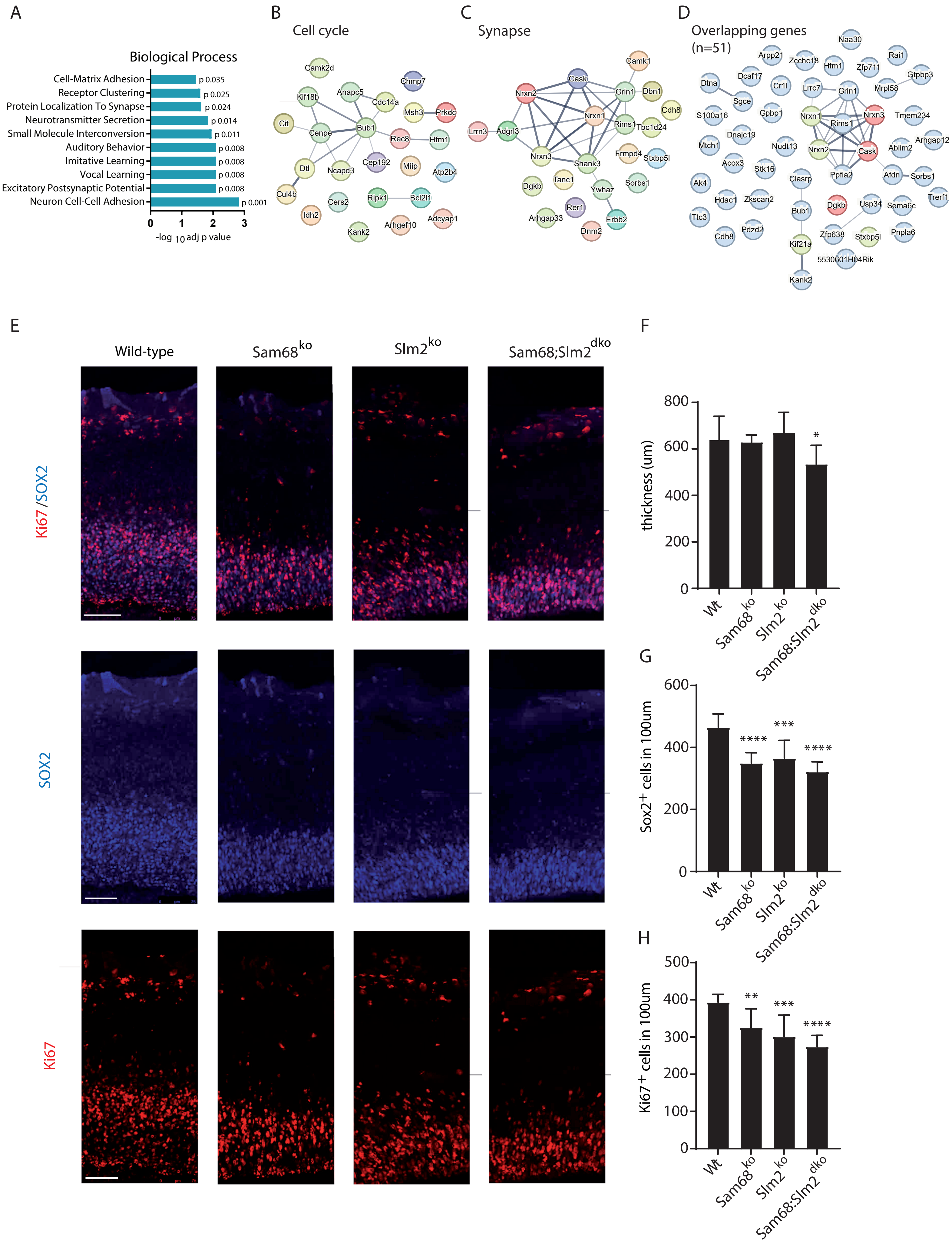

Supp. Figure 8

Supplementary Figure 8. Cortical developmental defects in *Sam68:Slm2*^{dko} mice. A) Gene Ontology (GO) analysis of the 51 genes overlapping with studies on single knockout mice. Bars length represents −log10 of adjusted p value. **B,C)** STRING analysis showing the network of cell cycle (B) and synaptic (C) genes differentially regulated in the *Sam68:Slm2*^{dko} cortex. The line thickness connecting the different genes/proteins corresponds to the strength of the supporting data. Disconnected nodes in the network are proteins whose interaction has not been determined due to lack of supporting data. **D)** STRING analysis showing the network of the 51 genes overlapping with studies on single knockout mice. **E-H)** Representative images (E) and quantitative analysis of the cortical thickness from ventricle to pial surface (F) or of neuronal cells stained for SOX2 (G) or Ki67 (H) in the cortex of wild-type, *Sam68*^{ko}, *Slm2*^{ko} and *Sam68:Slm2*^{dko} E15.5 embryos. Histograms show the data (mean + SE) of three independent male embryos for each genotype used for the analyses. Statistical analyses were performed using the one-way ANOVA test; *P<0.05; **P< 0.01; ***P<0.001; ****P<0.0001. (Scale bar = 75µm).

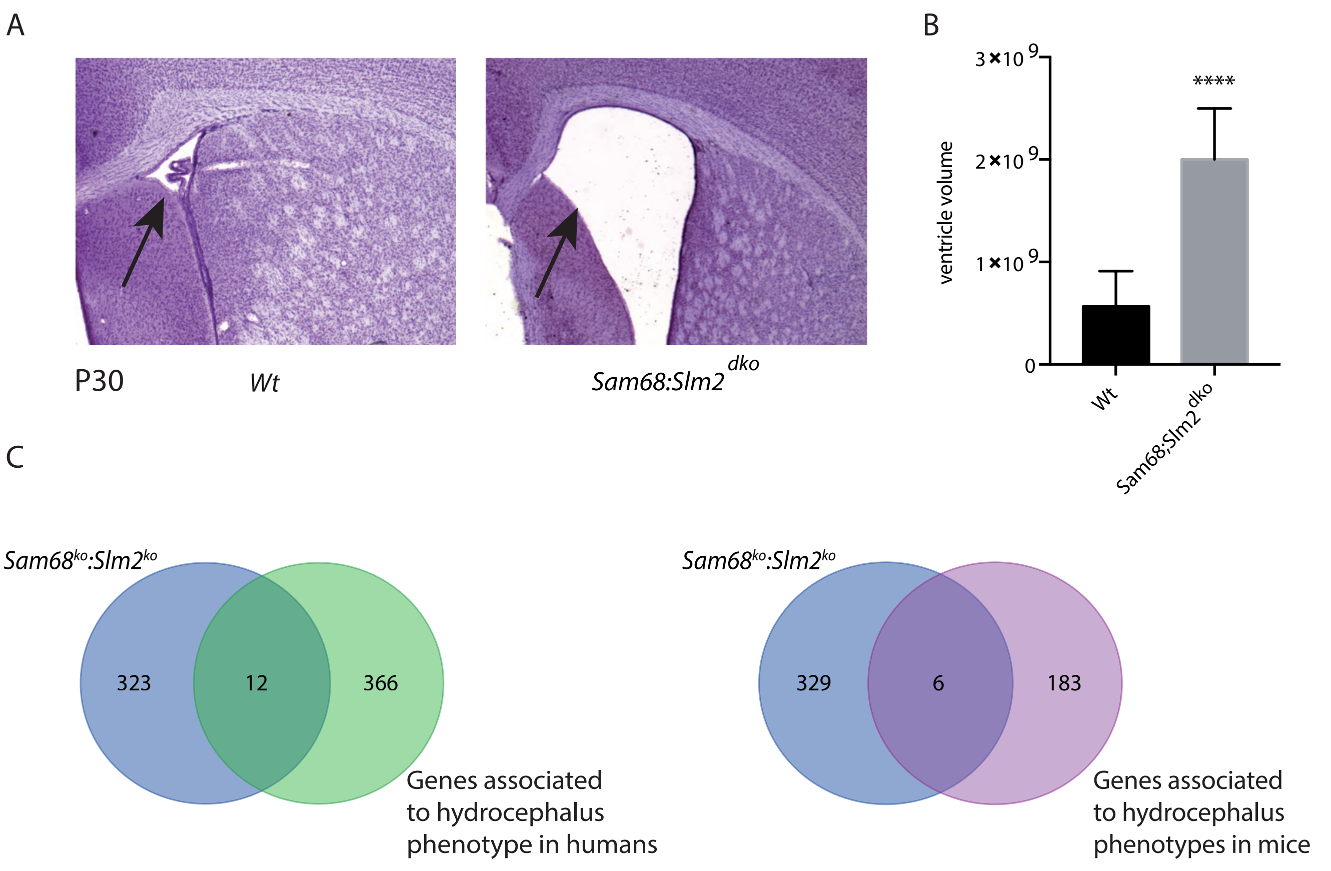

Supp. Figure 9

Supplementary Figure 9. *Sam68:Slm2dko* mice develop a hydrocephalus. A) Nissl-stained coronal sections of *wild-type* (*Wt*) and *Sam68:Slm2dko* age-matched mice showing a dilated lateral ventricle (indicated by arrows) in the *Sam68:Slm2dko* cortex. **B)** The bar graph shows the results of stereological quantitative evaluation of lateral ventricle volume (mean + S.E., n=4; Student's T test; $p<0.05$). **C)** Venn diagram showing the overlap between splicing events differentially regulated in the *Sam68:Slm2dko* cortex and the genes associated to hydrocephalus phenotype in humans (**A**) or mice (**B**).
